# Community Reaction to Bioterrorism: Prospective Study of Simulated Outbreak

**DOI:** 10.3201/eid0906.020769

**Published:** 2003-06

**Authors:** Cleto DiGiovanni, Barbara Reynolds, Robert Harwell, Elliott B. Stonecipher, Frederick M. Burkle

**Affiliations:** * National Naval Medical Center, Bethesda, Maryland, USA; † Centers for Disease Control and Prevention, Atlanta, Georgia, USA; ‡ Harwell Productions, Inc., Shreveport, Louisiana, USA; § Evets Management Services, Inc., Shreveport, Louisiana, USA; ¶ Johns Hopkins University Medical Institutions, Baltimore, Maryland, USA; # the Defense Threat Reduction Agency, Fort Belvoir, Virginia, USA

**Keywords:** behavior, bioterrorism, communication, Rift Valley fever, risk communication, terrorism, mass behavior, psychological, research

## Abstract

To assess community needs for public information during a bioterrorism-related crisis, we simulated an intentional Rift Valley fever outbreak in a community in the southern part of the United States. We videotaped a series of simulated print and television “news reports” over a fictional 9-day crisis period and invited various groups (e.g., first-responders and their spouses or partners, journalists) within the selected community to view the videotape and respond to questions about their reactions. All responses were given anonymously. First-responders and their spouses or partners varied in their reactions about how the crisis affected family harmony and job performance. Local journalists exhibited considerable personal fear and confusion. All groups demanded, and put more trust in, information from local sources. These findings may have implications for risk communication during bioterrorism-related outbreaks.

Human behavior during disasters (e.g., hurricanes, fires, mass shootings, airplane crashes) has been studied by historians as well as behavioral and social scientists, and disaster management teams make assumptions on the basis of these studies (*1*–*11*). However, with bioterrorism (intentional release of biological, chemical, or radiologic agents), the standard sensory cues (location, beginning and end of crisis) are not available; therefore, a different “emotional valence” may be involved. The standard models used as predictors of human behavior during crises may not be adequate. We simulated a bioterrorism-related outbreak in a U.S. community to examine (prospectively) the community’s reaction to the crisis and assess the need for public information.

## Methods

We simulated the intentional aerosolized release of Rift Valley fever virus (RVFV) in a semirural community (population 300,000) in the southern part of the United States. The community was selected because its mosquito population could support transmission of RVFV. We videotaped a series of simulated print and television (local, network, and cable) “news reports” over a fictional 9-day crisis period. The 83-minute videotape told the story of the intentional disease outbreak. We invited four groups (medical first-responders, medical first-responder spouses or partners, journalists, and others) within the selected community to view the videotape and answer questions about their reactions. These four groups knew that the outbreak was fictional. We then tabulated and analyzed the responses.

### The Video

The story of the simulated outbreak unfolded in a series of video reports from federal and local governments and the news media (Appendix 1). Health agency news bulletins were provided by the Centers for Diseases Control and Prevention; news reports by television reporters or news anchors; and community reports by local officials, including the mayor. The reports began with recognition in the community of an unusual infection affecting humans and certain farm animals and continued during the next 9 days with an epidemiologic investigation and the identification by federal authorities of intentional release of RVFV. Reports included a detailed press conference by federal health authorities describing routes of transmission, prevention measures, signs and symptoms of infection, and medical management of the disease. The news conference, held in the state capital the day after the presence of RVFV infection in the United States was announced, was immediately followed by a panel discussion (by nongovernment experts) on RVFV. Differences of opinion on clinical, epidemiologic, and biological issues among RVFV experts were reported.

Confusion arose in the community over disease management (e.g., the effectiveness of the antiviral drug ribavirin, the need for RVFV vaccine, and who should receive the vaccine) and over the potential for infected persons to serve as reservoirs and carriers of the virus elsewhere during the few days when viral titers are especially high. Governors of adjoining states questioned the adequacy of mosquito-control and animal quarantine measures, given the lack of a control model for the spread of RVFV infection in industrialized countries. Although official quarantine measures were not taken, final video reports showed a gradual de facto isolation of the city.

### The Questionnaire

The questionnaire (Appendix 2), which included multiple-choice, open-ended questions, and opportunities for additional comments, was distributed to all participants. Questions addressed job abandonment, quarantine compliance, demand for drugs and vaccine, information requirements, and other issues of community interest. Six sets of questions were posed to the participants during the video presentation. Set 1 was given after a disease of unknown etiology affecting humans and some farm animals was recognized in the community. These questions focused on willingness to remain at work, the types and sources of information that influenced the decision to work or not work, and actions regarding families and loved ones. Set 2 was given after the disease was identified as RVFV infection, federal health authorities briefed the public about this infection at a press conference, and a panel of nongovernment experts discussed the disease on television. These questions tested the participants’ understanding of RVFV routes of transmission and preventive measures and the participants’ satisfaction with information from government and nongovernment sources. Sets 3 and 4 followed a period of growing confusion and anxiety caused by changing and sometimes conflicting “authoritative” statements and tested participants’ requests for medication, including ribavirin (Set 3), and for RVFV vaccine (Set 4). Set 5, given after the participants learned that the outbreak of RVFV was intentional, reassessed decisions and actions regarding job and family concerns and information needed to make these decisions. Set 6 followed a period of increasing anxiety over a now-confirmed bioterrorism-related outbreak that could spread to humans and cattle in the state and in adjoining states, over the ability of the government to stop the spread of the infection, and over the de facto isolation of the community. These questions surveyed participants’ reactions to rumors of possible quarantine and to sources of information deemed reliable and influential in decision making now that the threat had become more complicated, personal, and disruptive.

### Participants

Four study groups, totaling 153 community residents, were formed. A goal of at least 30 participants per group was dictated by budgetary factors. The number of candidates contacted to assemble groups of at least 30 participants followed guidelines from marketing study groups (Harwell Productions, pers. comm.). One hundred thirty-eight medical first-responders (responders) were invited to participate in one study group; 58 responded to our invitation, 45 registered, and 38 attended. Reflecting the make-up of medical first-responders in the community, one third of the group’s participants were fire department emergency medical services personnel, and two thirds were emergency department personnel (nurses, physicians, technicians) from the area’s major medical centers. Eighty-three spouses or partners of responders (hereafter termed spouses) were invited to form a second study group; 47 responded, 44 registered, and 32 attended. Fifty-seven members of the local print and TV news media (hereafter, termed the media) were invited to a third study group; 50 responded, 42 registered, and 34 attended. Three hundred fifty invitations were sent to rank-and-file residents of the community (hereafter termed residents) to form a final study group. Twenty-three invitations were returned by the U.S. Postal Service as undeliverable; 73 responded, 46 registered, and 47 attended (1 registered participant failed to appear, but 2 invitees who had not registered arrived and participated).

Names of persons invited to participate in the first three groups were drawn blindly from rosters of eligible candidates prepared by their employers. Rosters of eligible candidates for the residents category were prepared from census tract data and cross-indexed telephone books (Table).

**Table:** Study group characteristics, simulated bioterrorism incident

	Responders (N=39) No (%)	Spouses (N=32) no (%)	Media (N=34) no (%)		Residents (N=48) no (%)
Sex					
Male	24 (61.5)	12 (37.5)	21 (61.8)		22 (45.8)
Female	15 (38.5)	20 (62.5)	13 (38.2)		26 (54.2)
Age					
18–26	7 (17.9)	6 (18.8)	4 (11.8)		4 (8.3)	
27–50	30 (76.9)	22 (68.8)	23 (67.6)	19 (39.6)		
51–65	2 (5.1)	3 (9.4)	6 (17.6)	11 (22.9)		
>65		1 (3.1)	1 (2.9)	14 (29.2)		
Family members in area						
Yes	38 (97.4)	31 (96.9)	22 (64.7)	44 (91.7)		
No			12 (35.3)	4 (8.3)		
No response	1 (2.6)	1 (3.1)				
Education						
Not high school graduate		1 (3.1)		1 (2.1)		
High school diploma/equivalency	7 (17.9)	8 (25.0)		6 (12.5)		
Some college	15 (38.5)	18 (56.3)	5 (14.7)	16 (33.3)		
College degree	13 (33.3)	3 (9.4)	25 (73.5)	16 (33.3)		
Post-graduate education	4 (10.3)	1 (3.1)	3 (8.8)	7 (14.6)		
No response		1 (3.1)				
Other			1 (3.0)	2 (4.2)		

Sex and age distributions within the four study groups were representative of the population segments from which their members were drawn, as were the educational levels of the responder, spouse, and media groups. The educational distribution of residents was not representative of the metropolitan area; those who chose to participate in this group had more formal education than area residents in general. According to 2000 census data for this area, 24.2% of the population have no high school diploma or equivalency, 30.2% are high school graduates, 22.4% have some college education, 12.7% have either an associate or full college degree, and 6.1% have graduate or professional degrees. We asked all participants if they had family members and loved ones in the area to assess potential conflict in job loyalty versus safety of one’s family and loved ones during a disaster. The media group was unique in the large number of its members who answered no to this question.

## Results

When asked about the transmission of RVFV, 30% to 35% of all group participants knew that the virus is transmitted by mosquitoes and not from person to person. RVFV transmission information was given by federal authorities at the televised press conference, as well as by academic experts interviewed on television after the press conference. The largest group who thought the risk for person-to-person transmission was “considerable” was responders. None of the participants was satisfied to receive information from federal authorities only. Most participants wanted additional information from local public health authorities, and 48% to 75% wanted information from both government and nongovernment sources.

In all four groups, participants who expressed interest in nonmedically indicated antibiotic or ribavirin treatment were in the minority. The largest minority to demand such medications was in the group of spouses; however, the demanded medication was primarily for their first-responder companions, not for themselves. In all four groups, approximately 50% of participants said that they would compete for RVFV vaccine for themselves and their families; the largest demand came from the media. When asked if they would demand vaccine for themselves as a quid pro quo for remaining on the job during the crisis, 26% of responders said they would, and 21% said they were uncertain. Sixty-three percent of spouses said they would want their mates to demand vaccine.  Reacting to rumors of quarantine (not to an official quarantine announcement), 59% of media and 75% of residents said they would comply and not try to leave; 6% of residents and 13% of spouses said they would try to leave regardless of consequences; 4% of residents and 15% of media said they would obey but try to leave if necessary. In all groups, most participants stated that their willingness to comply would increase if they were assured that quarantine was absolutely necessary and that it would work. In all groups, a majority faulted federal authorities for holding the joint press conference in the state capital, rather than at the scene of the outbreak 250 miles away.   Early in the simulated outbreak, before the disease was identified and its implications were known, pluralities in all groups wanted health information from local public health authorities. Responders wanted the information delivered within the chain of command at work; ranking second as the desired source of this information for all groups, except responders, was the private physician. After the disease was identified and terrorism was determined the cause, no single source of information at any level was chosen as desirable by any majority. Small pluralities in each group chose as the most reliable source of information “the head of the federal team working at the outbreak site,” “the President,” “a physician from a federal agency,” or “other.” When asked whom the participants considered most influential in decisions they would make about work, family, and themselves, once again no single authority figure emerged as the majority’s selection in any group. Selections with small pluralities were “family and loved ones,” “the President,” and “the head of the federal team working at the outbreak site.” Half the participants in all groups chose “other.” Within “other,” 34% of the participants chose another federal medical or nonmedical official, 4% chose state officials, 5% chose national (not local) media, 11% said they did not know at that time in the outbreak who would be most influential in their personal decision making, and 47% chose local leaders, including government and nongovernment officials.   The media indicated they would turn to a variety of local sources for their assignments. Most of these sources were professors in the local medical school, but other sources included the reporters’ personal physicians and veterinarians.  Most participants in all four study groups indicated they would remain on the job throughout the crisis. Initially, when the outbreak of an as-yet-unknown disease in the area was recognized, 97% of responders, 94% of media, and 77% of residents said they would remain at work; 11% of residents opted not to work, and the remainder said they were students, unemployed, or retired. Ninety-one percent of spouses said they would encourage their mates to continue working.  After the disease was identified and the outbreak was recognized as an act of bioterrorism, 95%, 71%, and 65% of responders, media, and residents, respectively, said they would continue working, and 78% of spouses said they would want their mates to remain on the job. In all groups, most participants said they would continue to work, provided that they received information about medical issues (particularly transmission and prevention), their work sites were adequately protected, and the community were unlikely to be exposed to another act of bioterrorism. Seventy-seven percent of media said that if their work put them at risk, they would expect their employers to provide protective measures (from insecticides to vaccine) and necessary medication and treatment.   Just before conclusion of the video, responders and spouses were asked how important it was to reach agreement with their partners on whether to stay at work, seek medicines, and send family members out of town. Twenty-six percent of responders said that concurrence would be essential, whereas 53% of spouses thought such agreement was essential.

### Discussion and Conclusions

The study was based on a simulated outbreak; therefore, the participants’ reactions were to a simulated, not an actual, crisis. For budgetary reasons, we could not recruit and compensate sufficient numbers of community residents to provide a statistical sample of the populations from which they were drawn; therefore, as with some forms of market research, participants’ answers to questions reflect their opinions and not those of their peers. Sample bias may have resulted from the absence of persons invited to participate who declined to do so; this may be especially important among residents, whose undereducated members were underrepresented. The choice of RVFV as the disseminated agent may have muted the responses of the participants because it has a relatively low death rate (1% to 10%) and does not have the high “fear factor” of some other diseases such as smallpox. Finally, the community we chose has its own customs, traditions, and ways of coping with a crisis that may not be shared by communities elsewhere.

Some participant reactions in this simulated-outbreak study are of particular interest. For example, disagreements between responders and spouses over reporting for duty during the crisis and demands for vaccine as quid pro quo for staying on the job could influence staffing levels and responder job performance in an actual bioterrorism-related outbreak. Risk communication messages may need to be crafted, tailored to the needs and concerns of first-responders and families, and delivered separately.

Journalists are key participants in risk communication (*12*–*16*), yet in this study, the media exhibited more fear than any group other than spouses, made high demands for vaccine, had the poorest understanding of medical issues associated with RVFV, and were most likely to stay away from work after terrorism was recognized. Had this been an actual bioterrorism-related outbreak, the media might not have served effectively as conduits of information to the public because they had not been adequately educated to eliminate confusion and dispel fear about their personal safety.

Participants in this study were not unique in their wariness of sole-source information, however authoritative or expert (*17*–*20*). Members of the actual community in which the simulated bioterrorism-related outbreak occurred wanted information from varied sources, even if the sources they mentioned differed in quality and reliability. Their reactions suggest that bioterrorism training should include information management for risk communicators and public affairs officers who have the responsibility of providing timely and accurate information to dispel the “fog” of rumors and misinformation present during the aftermath of an intentional disease outbreak. Journalists and other media specialists should participate actively in scenarios and other similar exercises to gain insight into the complexity of information management in a bioterrorism-related crisis.

As the simulated outbreak became more complicated and personally threatening, participants indicated a preference for information from local government and nongovernment sources or from federal officials at the outbreak site. Recognized, respected community leaders (e.g., private physicians, government and nongovernment officials) are likely to provide guidance in a bioterrorism-related crisis, just as they do in other crises. Training in the strategy and tactics of risk communication (*21*–*24*) should be expanded to include them. In an actual bioterrorism-related outbreak, these local leaders, supported by federal health authorities, should take the lead in communicating with local residents.

## Supplementary Material

Appendix 1 Note: The following text is from an outbreak simulation. These events did not occur.

Appendix 2 Note: The following questionnaire is from an outbreak simulation. These events did not occur.
