# Supplementary material for: Community Reaction to Bioterrorism: Prospective Study of Simulated Outbreak
Source: Emerg Infect Dis. 2003 Jun;9(6):708–12. doi: 10.3201/eid0906.020769 (PMC3001393; doi:10.3201/eid0906.020769)
Supplement: Appendix 1 — Note: The following text is from an outbreak simulation. These events did not occur. [file 02-0769-app1-s1.pdf]

Appendix 1(online only)

Note: The following text is from an outbreak simulation. These events did not occur.

Number 1—Week 1, Thursday, Local evening newscast

- *Open on black.*
- *Super the date: Thursday, Local Evening News*
- *Fade in to local television newscast with female Anchor seated at news desk delivering copy to camera with graphic over her shoulder.*

Anchor Dani Cates: (News Anchor delivering the closing lines of local story)

"Residents of several areas of the city are complaining *(Bring up audio on "city are")*. When contacted about the apparent increase in the number of parking tickets being written in the last month, the (City) Police Department had no comment."

- *Camera 2 takes the Anchor from a slightly different angle and starts a slow creep out.*

Anchor Dani: "Even though we're well into spring, some in (City) are suffering from an illness which usually strikes in colder weather.

Health Reporter Barbara Parsons has more."

- *Creep out is completed to reveal the health reporter seated next to the anchor.*
- *Cut to camera 1 with a shot of the health reporter alone. Super:* "News 11 Health Reporter Barbara Parson*" over bottom 1/4 of screen.*

(Graphic-'Late-Season Flu')

Health Reporter: "Our area is experiencing a number of cases of a flu-like illness that has people complaining of fever, aches and pains and extreme fatigue, although treating physicians say these patients don't have the runny noses and coughing generally associated with flu or other upper respiratory ailments. Public health officials are collecting information on the illness, but have yet to put a name to it, although they are sure that it's not encephalitis such as the outbreak that occurred in the (another) area last year.

"In any respect, people with this malady seem to do well with bed rest, plenty of fluids and Tylenol or Advil."

- *Cut to Camera 2 for two-shot of Anchor and Health Reporter.*

Anchor Dani: "Barbara, are there any precautions we can take against this illness?"

Health Reporter: "No Dani, the best advice seems to be, if you are experiencing these symptoms, go to bed, drink plenty of fluids, take Tylenol or Advil and see your doctor if you don't get better."

Anchor: "Sports is next after these messages."

Fade to black.

Number 2—Week 1, Friday, Morning, *The Times of (City)*

- *Fade in on black background.*
- *Super the date: Friday Morning*
- *Dissolve in mock-up of the front page of* The Times of (City), *—start creep-in, then dissolve to close-up of story on illness, below the fold, on the right side of the front page.*
- *Ghost out newspaper page and pull out until it is a background on which bullet points will be supered.*
- *Super headline, then bullet- point excerpts from the story, one at a time.*
- *Audio narration is delivered before headline and bullet points from the story are supered.*
- *Narration:* The following are points made in a news article appearing in the
- Friday, (date X) edition of *The Times of (City).*

- *To be supered—Narrator reads each point as it appears.*

"Mysterious illness grips (City)"
by Jake Reinhold

A growing number of residents of (City) are ill with some sort of flu or virus infection. Over the past 10 days local physicians have seen an increasing number of patients with some flu-like symptoms, fever, aches/pains and fatigue but without the coughing and upper respiratory difficulty associated with influenza.

Concerned by the number of cases of this illness, yesterday several local physicians met with local and state health officials to compare notes and to try and chart a common course of action.

The decision was made to send blood samples from 25 patients directly to the state health laboratory to speed identification of the disease.

Although they have not yet positively identified the illness, public health officials have assured *The Times* that this is definitely not an outbreak of encephalitis like the one that plagued the (another) area last summer and fall.

- *Fade to black.*

Number 3—Week 1, Friday, Local Evening News

- *Come up from black.*
- *Super the date: Friday, Evening News.*
- *Start the newscast from the very front with the lead bullet point then go to the opening of the newscast dissolve to the same local anchor, Dani Cates*
- *Anchor Dani Cates at news desk, super***—News 11 Dani Cates**

Anchor Dani Cates: "Yesterday we reported on an increasing number of cases of a flu-like illness in (City).

"News 11 has learned that local veterinarians are also dealing with strange health problems in the farm animals they serve.

"With more on the story, here's News II reporter Ted Brady."

- *Cut to videotape showing the vet at work with farm animals*
- *Video cuts to Ted Brady in farm lot doing stand-up for camera—super* **—News 11 Ted Brady.**

"For the last week and a half, local veterinarian Paul Silas has been seeing some peculiar symptoms in some of the farm animals he has been called out to treat."

- *Video cuts to head and shoulders of vet Paul Silas who speaks live on camera:*

"It's worrisome. Calves begin to vomit and have a pussy discharge from their nostrils. In two days they die. Pregnant cows abort. Adult cattle seem to be ill, but they recover. Horses don't seem to be affected, but on two different farms entire litters of puppies have died."

- *Back to Reporter Ted Brady on camera:*

"This morning several local vets met with physicians who have been treating patients with the flu-like illness that we reported on yesterday. At a later meeting with (City) health officials, it was decided to report their experiences to the state agriculture and public health offices."

- *Back to vet on camera:*

"Whether or not people and animals getting sick at the same time is a coincidence, we don't yet know, but I personally felt that we needed to look at this situation further."

- *Cut to Anchor Dani back in the studio*: "Right before air time, we asked local health officials if this could be another outbreak of encephalitis such as the one that occurred in (another city) last year. We were assured that even though this illness has not been identified, encephalitis has been definitely ruled out. Your News 11 team will stay on top of this for you and report developments as they occur."

- *Fade to black*

Number 4—Week 1, Saturday, Local Evening News

- *Come up from black*
- *Super date: Saturday, Evening News.*

- *Weekend Anchor delivers message with series of graphics over her shoulder:*

1) *"Mystery Illness"* ***2)*** *"CDC and USDA"*

· *Super over Anchor* **— News 11 – Phyllis Shaw**

Anchor Phyllis Shaw: "In our lead story tonight the uncertainty surrounding the mystery illness that seems to be affecting humans and animals in the (local state area) has prompted the state Public Health Service to request assistance from the federal government. That help arrived today as medical detectives from the U.S. Centers for Disease Control and Prevention and representatives for the United States Department of Agriculture's Animal and Plant Health Inspection Service arrived in the local area and immediately went to work trying to identify the malady. The team refused to talk with your News 11 team, but the Centers for Disease Control in Atlanta issued the following statement":

· *Cut from the Anchor to screened background on which the message is supered as it is read by the Anchor.*

· *This should also be a graphic.*

· *Centers for Disease Control and Prevention Statement is read*

"The (State) Department of Health has requested assistance from the Centers for Disease Control and Prevention in their investigation of cases of a flu-like illness occurring in the state. CDC epidemiologists *(disease detectives)* and laboratory scientists are assisting state and local public health officials in their investigation. Routinely, CDC assists state and local public health officials and health care professionals as they work to determine: 1) the cause of illness, 2) appropriate measures to reduce transmission of the disease, and 3) appropriate treatment for persons who become ill. Any announcements about this investigation will be released by the state health department. "

"As always our News 11 team will stay on top of this story reporting new developments as they occur."

- *Fade to black*

Number 5—Week 1, Sunday, *The Times of (City)* morning newspaper

- *Fade up from black.*
- *Super - Sunday, morning newspaper* The Times of (City)
- *Folded newspaper is shown against black, as camera starts slow zoom in.*
- *As newspaper fills screen and headlines are legible, cut to close-up of the portion of the paper that shows the story.*
- *Transform the newspaper into a ghost background on which type is supered*
- *Narrator reads the bullet points as they appear on the screen*

Lead Story:

THIS IS NOT THE FLU

CDC investigators seek answers to mystery illness.

Disease detectives from the U.S. Centers for Disease Control and Prevention arrived in the (City) area yesterday seeking to identify a mystery illness that is gripping (City).

Investigators from the United States Department of Agriculture are working along side the CDC personnel to determine if a deadly outbreak affecting area farm animals is linked to a flu-like illness that is on the increase in (City).

A survey of local medical facilities made by *The Times* showed that 124 cases of the illness have been confirmed with 6 to 8 deaths attributed to the disease. A spokesman for one hospital cautioned against considering *The Times* survey a definitive count, saying "many more who contracted the illness may have treated themselves rather than seek medical help."

Patients with the illness typically: have a fever up to 102 degrees; complain of extreme fatigue; show improvement after 5 to 7 days of bed rest, with plenty of fluids and treatment with medications such as Tylenol.

“Not all patients have the same experience,” cautions a physician who has treated multiple cases of the illness. The condition of some patients worsens as they develop liver problems and/or renal problems. In addition some patients develop confusion and may hallucinate as the disease involves their brain. **This is definitely not the FLU!**

- *After the completion of the first story, fade in another close-up of the paper that shows the headline: “(neighboring state) officials say their states not affected”.*
- *Make newspaper into background the crawl bullet points up the screen.*
- *Voice-over says:* “In the same edition and on the front page” *and then reads the following bullet points as they are supered on the screen.*

(sidebar)

(Neighboring states) OFFICIALS SAY THEIR STATES NOT AFFECTED

- In an attempt to determine the boundaries of the area being affected by the mystery flu-like illness, *The Times* contacted (neighboring states) public health agencies in counties bordering (state).
- Very much aware of the situation developing in the (city) area, these public health official have been closely watching their areas to see if an illness like the one in (City) develops in their respective areas.
- All public health officials in adjoining (states) counties report no incidence of an illness like the mystery illness in (City).

- *Fade to black.*

Number 6—Week 1, Sunday, Local Evening News

- *Fade up from black.*
- *Super-Sunday, Evening News.*
- *Lead local Anchor is on instead of weekend replacement.*
- *Super over Anchor*—**News 11–Dani Cates**

Anchor Dani: “The impact of the mysterious flu-like disease that seems to be on the increase in the (City) area, has been enormous in some local families.

“Health Reporter, Barbara Parsons, has the story.”

Barbara Parsons: (voice over video of widow seated in her home) “When Opal Wright’s husband Eugene turned up with a fever, neither she nor her husband thought much about it.”

Opal Wright’s on camera with tears

“Eugene, my husband, stayed home from work because he was so tired and he had fever. After two days, he was ‘acting funny’ and he didn’t know me or our son, Arthur.”

Barbara Parsons: (voice over video of emergency room at (local medical center) “Taken to (name) Med Center by his family, Eugene Wright was immediately admitted.”

Opal Wright - on camera - “The doctors, they really worked hard on Eugene, but nothing seemed to do any good. First, he got all yellow and Oh, - he threw-up so much - *(then she is in tears)* and then he began to bleed from his mouth and he just didn’t know us - and then my poor Eugene died.”

Barbara Parsons on camera in front of (medical center) – super over Barbara Parsons **– News 11 Health Reporter – Barbara Parsons:** “I contacted (name) Health Science Center officials about the death of Eugene Wright and was told that it is the policy of the Medical Center not to comment on individual cases where the cause of death is in question.

“Privately, other sources within the Medical Center told me that Eugene Wright’s unusual death is not an isolated occurrence, but one of several that have occurred recently.”

- *Cut back to Anchor.*

Anchor Dani: “With more than 125 cases of the mystery illness confirmed, the spread of the illness is causing concern in other areas.”

(While she is talking, the camera pulls out from head-to-waist shot to reveal the health reporter seated next to her.)

Anchor Dani: “News 11 Health Reporter Barbara Parsons is here with more on the story—Barbara …”

- As Parsons begins to speak, camera moves in on her to medium head and shoulders.
- Super: **News 11 Health Reporter – Barbara Parsons**

Health Reporter Parsons: “ Many lives are being impacted by the illness and death being experienced in (City). Here are the reactions of some of the people we’ve talked to.”

Video head/shoulders of man: “I’ve been watching it pretty close because I got a wife and two kids. I just wish they knew what it was.”

Video head/shoulders of older man: “I just trust in the Lord each day, because even if I knew what it was, there’s nothing I could do about it.”

Video head/shoulders of African/American woman: “I’m really scared. I know two people who got it and they still are really sick.”

Video head/shoulder of 20-year-old man: “Am I worried? Not me! Hey, that’s life. Two minutes from now I could step off this curb and get hit by a bus.”

Back to Health Reporter: “There are several groups that are becoming increasingly worried—the first responders, the emergency room personnel and the hospital personnel that deal with the ever-increasing flow of patients with this mystery disease. Is this illness contagious? At this time there’s simply not enough information developed to determine what’s being dealt with and whether or not it’s contagious. As one fire department EMT told me, ‘We’re professionals and we are going to do our job, but that doesn’t mean we aren’t concerned.’”—“Dani?”

Anchor Dani: “One final word. We contacted the state health department and as of twenty minutes before airtime, they had nothing further on their ongoing investigation.”

- *Fade to black.*

Number 7—Week 1, Sunday, Late Night Cable News

- *Fade up from black.*
- *Super-Sunday, Late Night Cable News.*
- *Fade into newscast with the Anchor delivering the last of another news story.*

WNN Headline News

"…….Officials of the National Weather Service expect the river to crest by midweek. No official damage estimates have been made by the counties involved. "

- *WNN Anchor delivers copy with graphics of CDC logo and (State) state map.*

"WNN Headline News has learned that investigators from the Centers for Disease Control and Prevention are in Northwest (State) studying a flu-like illness that seems to be on the increase in the (City), (State) metropolitan area. So far, there are 124 confirmed cases of the illness that have resulted in 6 to 8 deaths. U.S. Department of Agriculture investigators are also on the scene trying to discover if an unusual sickness affecting farm animals is linked to the outbreak in humans. No causes have been suggested for the outbreak."

- *Fade to black.*

Number 8—Week 1, Monday, National Network Evening News

- *Fade up from black.*
- *Super: Monday, Network Evening News.*
- *Medium close-up of Anchor with graphic over shoulder–"Bioterrorism in (State)?"*

UBC Anchor James Guest: "Has the United States suffered another terrorist attack? Officials of the Centers for Disease Control and Prevention are unwilling to rule out that possibility as their investigators work in Northwest (State) to identify the cause of an outbreak of a flu-like illness with nearly 150 reported cases. Six to eight deaths have resulted from the illness.

"United States Department of Agriculture personnel are also on the scene trying to determine if there is a link between the disease in humans and a sickness that has afflicted area farm animals.

"In answer to questions posed to them about the causes of the outbreak in (State), the CDC in a statement released today said:

'One question that CDC disease investigators routinely ask as part of their investigation is, "Could this outbreak have been caused intentionally?" CDC investigators will maintain open minds as data in this investigation are collected and analyzed. We must caution, however, that it is not in the best interest of public health to speculate on its introduction until we have the data necessary to put forward a theory. CDC also cautions that despite our best efforts to answer this important question, we may never have a definitive answer. Any specific questions regarding bioterrorism as part of this outbreak investigation must be answered by the FBI and law enforcement officials.'"

- *Fade to black.*

Number 9—Week 1, Tuesday, Local Evening News

- *Fade from black.*
- *Super-Tuesday, Local Evening News.*
- *Open with Anchor at desk with graphic over her shoulder–super*–**News 11–Dani Cates**.

Anchor Dani: “What it is remains a mystery, but the concerns of virtually everyone in (City) and a growing number of people across the nation are focused on the mysterious flu-like illness that seems to be tightening its grip on the local area. More than 135 cases of the illness have been confirmed and 6 to 8 deaths have been attributed to the ailment.

“A concurrent sickness has also ravaged the farm animal population in the area. With little known about the illness and not even a name by which to call it, how is (City) coping? News 11 Health Reporter, Barbara Parsons, takes a look.”

(Pull back to show both the Anchor and Health Reporter in two-shot.)

Health Reporter Parsons: “Dani—how you’re dealing with this mystery illness depends on how close you’ve come to the sickness. Our News 11 team offers some insights.

“I’ve been on the front lines, so to speak, with the first responders, the Fire Department EMT crews who have transported many of those with the illness to the emergency rooms in (City).”

Video of EMT fireman: Audio: “We just wish we knew what it was-that’s what’s tough—not knowing. But, in a situation like this, you fall back on your training and take every precaution you can.”

Health Reporter Barbara Parsons: “Dealing with disasters is the job of Civil Defense Director (name). (Name), are we dealing with a disaster?”

(Camera tightens on [name]) “With less than 150 confirmed cases of this illness, this doesn’t qualify yet as a disaster for the entire community, only to those who have become very sick or the relatives of those who have lost a loved one. Hopefully, this illness will be identified very soon and our emergency network will not have to be activated.”

Health Reporter Barbara Parsons: “A disaster is exactly what’s happened to Brittany Wilson, age 8, whose family lives on a (name) Road farm.

(Cut to tape of Brittany)-“My golden retriever, Bonnie, had her first litter of puppies 3 weeks ago and they were so beautiful. Then last week, my dad’s calf got sick, then, just like that the puppies got sick. Now they’re all dead and Bonnie just wants to lie there. I miss my puppies so much.”

Cut back to Health Reporter Parsons: “What is it that shows no regard for humans and farm animals? Hopefully, the Centers for Disease Control will soon put a name on it and (City) residents will know the enemy that is stalking the area.”

- *Fade to black.*

Number 10—Week 1, Tuesday, Late Evening Local News, 10 p.m.

- *Fade in to black.*
- *Super-Tuesday, Late Evening Local News.*
- *Bring up video of local news anchor, Dani Cates, sitting at the news desk.*

News Anchor Dani Cates: “Good evening ladies and gentlemen. Before we begin our local report, we take you to UBC studios in Washington, DC, for a special report.”

(Cut to graphic - UBC Washington - dissolve to special report Anchor)

- *Super*–**UBC News–James Guest**

Anchor Guest: “Good evening. I’m James Guest of UBC News in Washington. The White House, at a hastily called press briefing, has issued a statement of great significance. For more we go live to UBC White House correspondent, Faye Ray.

- *Cut to shot of Fay Ray in front of the White House.*
- *Super*–**UBC White House correspondent Faye Ray**

Correspondent Fay Ray: “James, White House Press Secretary Ari Fleisher has just issued a statement that deals with the mysterious outbreak of a disease in Northwest (State). I quote from the statement”:

‘*Responding to requests from health officials in (State), scientists from the Centers for Disease Control and Prevention and from the U.S. Department of Agriculture have for the last 72 hours been investigating the outbreak of a disease in Northwest (State) that has affected both humans and certain farm animals. They have determined that the cause of this disease is called Rift Valley fever virus, which is transmitted primarily by mosquitoes. This is the first known outbreak of this infection in the United States. At this time, investigations are continuing into the origins of the outbreak in Northwest (State). Appropriate agencies of the federal government are working with local and state officials to control and eradicate this virus.’*

Faye Ray: “So James, there you have it. Northwest (State), specifically the area including and surrounding (City), (State), is the scene of an outbreak of Rift Valley fever, an illness heretofore found only in sub-Saharan Africa and the Middle East.”

Anchor James: “Faye, was there anything else?”

Faye Ray: “The White House statement also said that further information about this disease is being issued by the CDC. Health experts from the CDC, the USDA and the (State) Departments of Health and Agriculture will hold a press conference in (state capital) tomorrow starting at 8:00 a.m. (x) Daylight Time.”

Anchor James: “Faye, what else did Ari Fleisher say about the Rift Valley fever outbreak?”

Faye Ray: “James, Ari Fleisher took no questions and referred us to the statement he issued and the press conference to take place in (state capital).”

Anchor James: “The statement refers to investigations continuing into the origins of the outbreak. Do they suspect this is an act of terrorism?”

Faye Ray: “We don’t know, James. Again, Ari Fleisher took no questions, referring us to the statement and tomorrow morning’s press conference.”

Anchor James: “Thank you, Faye. The Centers for Disease Control has just issued a statement on the Rift Valley fever outbreak in (State).

UBC correspondent, “Terry Vetch has the CDC statement. Terry?”

- *Cut to another correspondent, Terry Vetch, in studio to read statement.*

Terry Vetch: “James, the statement was issued by the CDC just moments ago. I quote”: *For the past few weeks, a disease has been affecting both people and certain farm animals. Responding to requests from state health officials in (State), scientists from the Centers for Disease Control and Prevention and from the U.S. Department of Agriculture have, for the past 72 hours been investigating the outbreak of a disease in Northwest (State) that has affected both humans and certain farm animals. They have determined that the cause of this disease is called Rift Valley Fever virus, which is transmitted primarily by mosquitoes. This is the first known outbreak of this infection in the United States. At this time, investigations are continuing into the origins of this outbreak in Northwest (State). Appropriate agencies of the federal government are working with local and state officials to control and eradicate this virus. Further information about this disease is being issued by the CDC, the U.S. Department of Agriculture and the (State) Office of Public Health at a press conference to be held in (State capital), (State), tomorrow morning at 8:00 a.m. (x) Daylight Time.’* (Bullet points are supered on the screen.)

Anchor James: “Terry, the statement makes no mention of how the disease appeared in Northwest (State).”

Terry Vetch: “No, it doesn’t James. I assume we’ll just have to wait for further information from the CDC and law enforcement agencies that are still investigating the origin of this outbreak.”

Anchor James: “Thank you, Terry.

“To recap-The White House has just announced that the disease outbreak in Northwest (State), specifically the (City), (State), is Rift Valley fever, the first such occurrence in the United States. Rift Valley fever is a viral infection spread by mosquitoes that has been confined primarily to sub-Saharan Africa.

“In Northwest (State) there have been some 150 confirmed cases of the disease with 6 to 8 deaths.

“We have just received word that (State) Governor (name) has mobilized units of the (State) National Guard for mosquito eradication in the (name) area of Northwest (State). The first Guard units are expected in (City) sometime tomorrow.

“To repeat, (State) Governor (name) has mobilized units of the (State) National Guard to eradicate mosquitoes in Northwest (State) where 6 to 8 deaths and 150 cases of Rift Valley fever have been confirmed. Rift Valley fever is African in origin and is spread by mosquitoes.

“We now return you to regular programming.”

- *Fade to black.*

Number 11—Week 1, Wednesday, Early Morning

from (State Capital)

Special Report from UBC news with the Anchor in Washington

- *Fade up from black.*

- *Super: "Wednesday, Early morning news conference from (State capital), (State).*
- *The segment opens with special report header:* **Outbreak in America** *graphic with map of U.S. with (affected region) highlighted and with the audio and graphics: "***A special report from UBC News, Outbreak in America.**

(Audio) "Now from the UBC newsroom in Washington, here is UBC News Correspondent, James Guest."

- *The setting is a tight head to waist format so that graphics show over left shoulder with a banner across the bottom 25% of the screen,* **"Outbreak in America."**
- *As the anchor mentions "Northwest (State)", bring in graphic over his shoulder that shows map graphic of the area with language "***Rift Valley fever***"*.
- *Next graphic up reads, "***Rift Valley fever—150 confirmed cases—6 to 8 deaths***"*

Anchor James: "Good morning!...scarcely 12 hours has passed since scientists of the United States Centers for Disease Control and Prevention confirmed that a deadly virus heretofore not found in the United States is now present in (State). In an unusual late-night announcement from the White House, it was confirmed that the mysterious outbreak of disease in Northwest (State) that has effected both humans and certain farm animals is Rift Valley fever, a viral infection primarily transmitted by mosquitoes. So far approximately 150 confirmed cases of the disease have been identified with 6 to 8 deaths attributed to the illness.

"This outbreak is the first known instance of the disease in the United States. With its origin being sub-Saharan Africa, the disease has been located primarily on that continent, with an outbreak occurring in Yemen and Saudi Arabia in 2000. The White House statement promised more concrete information on the outbreak at a news conference with CDC, USDA and (State) Health and Agriculture officials to be held at this hour in (State capital), (State). Members of the press are gathered in the Governor's conference center in the (State) State Capitol Building awaiting the start of that news conference. One of the topics that's sure to be explored is how the RVF virus was introduced into the (City) area of Northwest (State).... we're being told that the press conference is about to start....."

- *Cut to medium shot of stage with two short tables and a table- top podium in the middle. The tables are draped with white cloths with blue skirts. Podium has logo of the State of (State) on it. After the scene is established, the "***Outbreak in America**" swipe *across the bottom 20% of the screen is established, with the language "***(State capital), (State)***."*
- *As the scene is established, Several men and a woman make an entrance on the stage from the right of the picture*
- *Those who have entered sit at the tables on the stage and woman comes to the podium.*

(Name): "Good Morning. I am (name), Assistant Secretary of the (State) Department of Health. We are here to discuss the disease outbreak in the (City) area of Northwest (State)."

(Name): "For those of you in the television audience who may not be familiar with (State), (City) constitutes a metropolitan area in the northwest corner of our state about (x) miles east of the border with (name of neighboring state) and (x) miles south of the (name of another neighboring state) line. With a population of some 350,000 plus, the area has significant farming and cattle interests, manufacturing and gaming and it is the home of (a place of interest)."

(Name): "During the week of (dates XXX), alert physicians and hospital officials identified a significant number of patients suffering a similar flu-like illness. After consultation, they enlisted the assistance of the (State) Department of Health in diagnosing the illness. During this same period, veterinarians in the (City) area were dealing with an unusual sickness affecting farm animals. After comparing information, area physicians, veterinarians and state health officials requested the assistance of the Centers for Disease Control and the U.S. Department of Agriculture's

Animal and Plant Health Inspection Services. Investigators from both agencies were immediately dispatched to the (City) area to start the investigation that led to yesterday's declaration by both CDC and the Department of Agriculture that Rift Valley fever had entered the United States."

(Name): "Here with us today are Dr. Forrest Daly of the Centers for Disease Control and Prevention, Special Pathogens Branch and Dr. Peter Cobb of the Veterinary Services Unit of the United States Department of Agriculture's Animal and Plant Health Inspection Services. Today we begin first with Dr. Daly."

Dr. Daly: "Good Morning. I want to start by commending health officials in (name of counties) in Northwest (State) for their actions in this situation. Their immediate response to this outbreak and their assistance in identifying this virus has been exceptional. The (State) Office of Public Health's prompt actions and the invaluable assistance they've given the scientists of the Centers for Disease Control and the USDA's Animal and Plant Health Inspection Services, have been a key part of the effort to quickly identify this outbreak."

Dr. Daly: "As you know, last evening scientists from both the Special Pathogens Branch of the CDC and USDA's Animal and Plant Health Inspections Services laboratories, confirmed that the outbreak of illness that has affected both humans and certain farm animals in Northwest (State), specifically the (City) area, is Rift Valley fever. These are the first reported cases of Rift Valley fever disease in the Western Hemisphere. Rift Valley fever is a viral disease transmitted primarily by mosquitoes. Even though this is the first reported instance of the disease in the United States, the CDC and other federal agencies have had ample opportunity over the past few decades to study the RVF virus in the laboratory and in Africa, where it is endemic, and in Saudi Arabia and Yemen, where an outbreak occurred in 2000. CDC, the (State) Office of Public Health and the local health units in the affected area of Northwest (State) have a great deal of experience with diseases transmitted by mosquitoes and there is a system in place to begin accelerated mosquito control measures in the affected areas.

Dr. Daly: "Now we understand that persons living in the affected areas are greatly concerned about their health and the health of their children and families. So CDC recommends these practical measures that are effective in situations such as this one.

- *As Dr. Daly mentions the points they are supered on the screen.*
    - People in the affected areas should avoid outdoor activities during the early morning and evening hours when mosquitoes are most likely to bite
    - If you must be outdoors, wear an insect repellent that contains DEET; you should also wear long-sleeved shirts and pants if your are outdoors.
    - Make a survey or your surroundings and eliminate all standing water if possible, including pet water bowls, flower pots, birdbaths and stagnant pools.
    - If you develop flu-like symptoms which include fever, generalized weakness, back pain, dizziness, and extreme weight loss at the onset of the illness, then you should immediately seek medical care and the supportive therapy that is the appropriate treatment for this disease.
- Those are the practical things that we all should and can do. I am now going to give you more specific information and its implications.
    - Rift Valley fever (RVF) is an acute, fever-causing viral disease that affects domestic animals (such as cattle, buffalo, sheep, goats, and camels) and humans.
    - In other parts of the world, RVF is most commonly associated with mosquito-borne epidemics during years of heavy rainfall.
    - Humans can get RVF as a result of bites from mosquitoes. Humans can also get the disease if they are exposed to either the blood or other body fluids of infected animals.
    - RVF virus can cause several different disease syndromes. People with RVF typically have either no symptoms or a mild illness associated with fever and liver abnormalities.

  - In some patients the illness can progress to hemorrhagic fever (which can lead to shock or hemorrhage), encephalitis (inflammation of the brain, which can lead to headaches, coma, or seizures), or ocular disease (diseases affecting the eye). So you see symptoms of the disease are quite broad.
  - Typically, patients recover within two days to one week after the onset of the illness. The most common complication associated with RVF is inflammation of the retina (a structure connecting the nerves of the eye to the brain). As a result, approximately 1% to 10% of affected patients may have some permanent vision loss.
  - Approximately 1% of humans that become infected with RVF die of the disease.
  - There is no established course of treatment for patients infected with RVF virus. However, studies in monkeys and other animals have shown promise for Ribavirin, an anti-viral drug, for future use in humans. Additional studies suggest that interferon, immune modulators, and convalescent-phase plasma may also help in the treatment of patients with RVF.
  - Fatality rates are significantly higher for infected animals. The most severe impact is on pregnant livestock infected with RVF, where virtually 100% of fetuses are aborted.
- As I said earlier a person’s chances of becoming infected can be reduced by taking measures to decrease contact with mosquitoes and other blood-sucking insects. Avoiding exposure to blood or tissues of animals that may potentially be infected is also another important protective measure for persons working with animals in RV-endemic areas.

“So what can be done to address the threat of RVF?”

- Vaccines for veterinary use are available, but they can cause birth defect and abortions in sheep and they induce only low-level protection in cattle.
- The human live attenuated vaccine, MP-12, has demonstrated promising results in laboratory trials in domestic animals.
- The close monitoring of human and animal populations for RVF virus infection is essential to learning more about how RVF virus infection is transmitted and to formulate effective measures for reducing the number of infections. Thank you for your attention.”
- *Dr. Daly completes his presentation and moves to take his seat as (name) returns to the podium.*

(Name): “We’ll complete the statements before we entertain questions. Dr. Peter Cobb of the Veterinary Services Unit of the USDA’s Animal and Plant Health Inspection Services, will discuss other aspects of the disease and its effect on humans and animals. Dr. Cobb....”

Dr. Cobb: “RVF was first reported among livestock by veterinary officers in Kenya in the early 1900s. RVF is generally found in regions of eastern and southern Africa where sheep and cattle are raised. However, RVF virus also exists in most countries of sub-Saharan Africa and Madagascar. RVF virus primarily affects livestock and can cause disease in a large number of domestic animals. (This situation is referred to as an ‘epizootic.’)

The presence of an RVF epizootic occurrence can lead to an epidemic among humans who are exposed to diseased animals. The most notable epizootic of RVF, which occurred in Kenya in 1950-1951, resulted in the death of an estimated 100,000 sheep. In 1977, the virus was detected in Egypt. It was probably exported there in infected domestic animals from Sudan. The result was a large outbreak of RVF among animals and humans. The first epidemic of RVF in West Africa was reported in 1987 and was linked to the construction of the Senegal River Project. The project caused flooding in the lower Senegal River area and altered interactions between animals and humans resulting in transmission of the RVF virus to humans.

An epizootic of RVF is generally observed during years in which heavy rainfall and localized flooding occur. Excessive rainfall allows mosquito eggs, usually of the genus Aedes, to hatch. The mosquito eggs are naturally infected with RVF virus, and the resulting mosquitoes transmit the virus to the livestock on which they feed. Once the livestock is infected, other species of mosquitoes can become infected from the animals and

can spread the disease. It is also possible that the virus can be transmitted by other biting insects. Thank you."

- *Dr. Daly returns to the podium.*

Dr. Daly: "We will soon be issuing detailed guidelines to healthcare workers who provide medical care to patients with Rift Valley fever, but in summary, there is no danger to physicians, nurses and other medical personnel as long as they use standard barrier precautions. Patients can be treated safely in regular hospital rooms if windows are shut or screened.

- *(Name) returns to the podium as Dr. Peter Cobb takes his seat.*

(Name): "We'll now take a limited number of questions. There in the second row."

First off-camera question: "This question is for Dr. Daly of the CDC. Doctor, in your statement you said that Rift Valley fever was first detected in livestock in Kenya in the early 1900s, nearly 100 years ago, yet this outbreak in North (State) is the first known outbreak of the disease in the U.S. Could the introduction of RVF be a terrorist act?"

Dr. Daly: "Actually, that 'early 1900s date' was mentioned by Dr. Cobb of the USDA, but I'll go ahead and answer your question."

Dr. Daly: "Investigations are continuing into the origins of this infection and at this time there is no reason to rule in or rule out bioterrorism. However, I want to point out that RVF has occurred naturally in Africa and elsewhere and could be introduced naturally in the United States just as the West Nile Fever was introduced into the U.S. naturally several year ago."

- *(Name) calls on the next questioner.*

(Name): "There in the third row."

Second off-camera question: "Are there any indications that this outbreak has spread to other geographic areas?"

Dr. Daly: "I'll take this one, too. We have received no reports that would suggest the presence of the Rift Valley fever virus anywhere beyond the (City) metropolitan area."

(Name): "There in the back." *(as she points off camera)*

Third off-camera question: "Since this disease primarily effects farm animals, is quarantine under consideration?"

- *Dr. Cobb of the USDA moves to the podium.*

Dr. Cobb: "Effective control measures are being developed to ensure that this disease does not spread beyond the (City) area. The first measure of course, is more effective mosquito control. Whether or not other measures are needed will be determined at a later date."

(Name): "Thank you very much ladies and gentlemen."

- *Those on the stage exit, ignoring the further questions from the press.*
- Fade to black.

Number 12—Week 1, Wednesday, After the (State capital) News Conference

- *Fade up from black.*
- *Super: Wednesday, UBC network special coverage.*
- *This segment takes place immediately after the conclusion of a news conference held in (State capital), (State), that discussed the RVF outbreak in Northwest (State). UBC anchor, James Guest, is the host.*
- **Outbreak America** — *graphic across bottom of screen.*

Anchor James Guest: "You have just witnessed a press conference held in (State capital), (State), concerning the outbreak of Rift Valley fever in the (City), (State), in the northwest corner of the state.

"Experts from the Centers for Disease Control and Prevention's Special Pathogen's Branch and the United States Department of Agriculture's Animal and Plant Health Inspection Services discussed the origins and characteristics of the disease, as well as the fact that both agencies believe the disease is spread to humans by mosquitoes.

"Questioned on how the virus was introduced into the United States, the representatives of both agencies were unwilling to rule out bioterrorism as the cause, saying, and I quote: 'Investigations are continuing into the origins of this infection and at this time there's is no reason to rule in, or rule out bioterrorism.'

"Joining me now are three experts in this field, who can help us understand the dimensions of the problems to be faced with an infestation of this type."

- *As each 'expert' is introduced, the screen is split so that we end up with four head/shoulders on the screen. As each answers a question or makes a point, we cut to a single shot of that individual.*
- *The name and title of each is supered briefly as they are introduced and as they begin remarks.*

Anchor James Guest: " Dr. Amos Labor is a professor of Medicine at the Harvard Medical School and the chief of Infectious Diseases at Massachusetts General Hospital in Boston.

"Dr. E.B. Decker is a professor of Medicine at the University of Texas Medical Branch at Galveston, Texas, and also a virologist.

"And, Colonel Bryan Rite, U.S. Army, retired, is a virologist and the former chief scientist at the U.S. Army Medical Research Institute of Infectious Diseases at Fort Detrick, Maryland."

Anchor James Guest: "Dr. Decker,....let's begin with you. Did the CDC and USDA people paint an accurate picture of the disease?"

Dr. E.B. Decker: "Well..., yes, as far as they went, theirs was an accurate picture. Rather simplistic, if I may say so, but accurate."

Anchor James Guest: "Is there other information that you feel should have been added?"

Dr. E.B. Decker: "How the disease is spread is of primary importance and, yes, it is spread by mosquitoes. But it can also be spread by aerosol."

Anchor Guest: "By aerosol, I take it you're referring to an airborne spread of the virus."

Dr. E.B. Decker: "Yes, you are correct. Veterinarians in particular can get it that way. They may come in contact with sprays of blood as they work on infected animals and can inhale some of the blood. Several published articles build a strong case that RVF can be acquired by inhalation."

Col. Rite: "I agree. There's ample evidence to support the theory of Rift Valley fever infection being spread by inhalation, as well as by mosquitoes. Not only humans but puppies and kittens have also been shown to acquire the infection by inhalation and ingestion. And I know that both of my colleagues are familiar with our work on the Egyptian strain of RVF and its 'respiratory infectivity.'"

Anchor Guest: "Then a person could get it from another human through coughing or even sneezing"

Dr. Decker: "No! There is no evidence to suggest this."

Anchor Guest: "According to the CDC's special pathogens website, RVF is considered one of the hemorrhagic viruses like Ebola, isn't it?"

Dr. Decker: "Well, yes, technically it's classified as one of the hemorrhagic fevers."

Anchor Guest: "Well, if it is considered one of the viral hemorrhagic fevers, let me read you a passage from a paper published in 1996 in the Archives of Virology."

'Most of the viral hemorrhagic fevers are caused by viruses that are handled in high containment laboratories in Europe and the United States because of their pathogenicity, or dangerousness and their aerosol infectivity. Special precautions should be taken when caring for patients infected with these viruses, but most hospitals can safely provide high quality care. The major danger is parenteral inoculation of a staff member (pause...) that is, a healthcare worker accidentally sticking himself with an infected needle ... *Fomites and droplets must be considered as well. The role of small particle aerosols in inter-human transmission continues to be controversial.* Note, that it says controversial, not impossible. *We believe that the aerosol infectivity observed for these viruses in the laboratory and the rare clinical situations that suggest aerosol spread dictate caution.'*

Anchor Guest: "One can't help but notice the contradiction between the CDC's position that people aren't infected through exposure to sneezes and coughs of other people, and this information.

"Dr. Decker, can you be sure that one human can't get the virus from another human through coughing or sneezing?"

Dr. Decker: "As I said before, Mr. Guest, there is no evidence to support that form of transmission. Furthermore, you should have noticed that I was one of the authors of the paper you just cited. The rest of the abstract, which you did not read, said that there have been **no** instances of person-to-person transmission through coughing or sneezing among patients and doctors and nurses."

Dr. Labor: "Yes, I agree with Dr. Decker. This virus has certainly not been studied as well as some of the more common viruses, but where we have looked at it, we've certainly seen absolutely no evidence of human-to-human transmission."

Anchor Guest: "Let's move from theory to practice. In Northwest (State), we already have some 150 confirmed cases of RVF and 6 to 8 deaths. Emergency medical people have been involved, emergency rooms also and doctors and nurses and various other hospital workers. Are you saying that all these people are safe?"

Dr. Labor: "As far as we know, they are. As the CDC statement accurately said, medical workers can safely treat these patients if they use standard barrier precautions."

Anchor Guest: "Doctor, you mentioned CDC. When anthrax infected people in buildings in Florida, New York, and New Jersey and Washington, CDC scientists offered initial advice and guidance that they later had to modify. I'm sure you would agree that anthrax has been a better-studied germ than the RVF virus. If CDC made those mistakes in early guidance on anthrax, how can we be sure the CDC is right in its guidance to healthcare workers about RVF?"

Dr. Labor: "You are taking the occurrence of anthrax and all of what happened during that situation and trying to apply it to a different disease and totally different set of circumstances and make a value judgment. That's simply not possible."

Col. Rite: "Well, wait a minute. Hospital lab workers may have to take some special precautions. Like vets, they handle blood and infected tissue where the virus can be present in concentrated form. Certainly in our military research laboratories, people who work with this virus in concentrated form do so under special conditions that we call Biolevel 3 and are even protected by RVF vaccine."

Anchor Guest: "There's an RVF vaccine?"

Col. Rite: "The vaccine that I referred to is an experimental vaccine that is classified by the FDA as an Investigational New Drug and the rules for its use are too strict and complicated for widespread use."

Anchor Guest: "We need to wrap this up, but there are two other questions I want to ask while our experts are with us. Dr. Decker, if mosquitoes transmit this infection from animals to people, could mosquitoes also transmit the infection from people to animals and people to people?"

Dr. Decker: (pauses) "Well, theoretically, that is possible—there is a window of about 3 or 4 days when people infected with the virus have enough of it in their blood so that if they are bitten by a mosquito during this time, that mosquito could theoretically carry the infection elsewhere to another person or animal."

Anchor Guest: "And finally CDC has said that about 1% of those who get infected with the RVF virus, die—but we already have a higher mortality rate in Northwest (State) and this infection may just be beginning. Why is the death rate so high in (State)?"

Col. Rite: "The overall mortality rate as the CDC mentioned in Africa over the years, has been one percent. However, in Yemen from August to November in 2000, there were nearly 1100 case patients and 121 deaths. That was an 11% mortality. We have to keep in mind that we've studied this disease mostly in Africa and sub-Saharan Africa at that. We may not know how this disease acts under conditions where records are better kept."

Anchor Guest: "Thank you, Doctor. We'll return to 'Outbreak in America' after these messages."

- *Fade to black.*

Number 13—Week 1, Wednesday, 1:30 p.m. local newscast

- *Open on black.*
- *Super the date: "Wednesday, 1:30 p.m."*
- *Fade in to local television newscast with female anchor seated at news desk delivering copy to camera with graphics over her shoulder.*
- *Graphic is* **"Outbreak in America."**

Anchor Dani Cates: “Thank you for remaining with us as we follow, “Outbreak in America,” the Rift Valley fever outbreak that’s gripping (City).

“It has been less than 16 hours since the disease that’s been on the steady increase in (City) was identified as Rift Valley fever, a viral infection that is spread by mosquitoes. At least that’s what the Centers for Disease Control has assured us.

- *Cut to video clip with Dr. Daly of CDC saying the disease is spread by mosquitoes.*

Anchor Dani: “Since that explanation at a joint press conference of the CDC, USDA and state health officials held in (State capital) this morning, other medical experts have questioned that premise.”

- *Cut to video clip with other expert saying the virus can be spread by humans and spread by aerosol.*

Anchor Dani: “Those differing assertions on how the disease is spread may be responsible for what we’re seeing on the ground here in (City) where theory takes a back seat to the practical. Here’s News 11’s Health Reporter Barbara Parsons with more. Barbara….”

- *Cut to Barbara Parsons in front of (name) Hospital*
- *Super*: **News 11 Barbara Parsons**

Barbara Parsons: “Dani we have contacts at every major medical facility in the metro area and though none of them will go on camera with us ... they are all saying the same thing......a number of healthcare workers, primarily those who are scheduled to report for the 3:00 p.m. shift, are calling in to say that they will not report for work. They give a variety of reasons. Some say they’re not well themselves, others cite illness in the family or a need to make a quick trip out-of-town. The few that will admit it, say that they are scared, given the growing controversy over how RVF is spread.”

Anchor Dani: “Is this concern confined to hospital workers?”

Barbara Parsons: “No, Dani. While checking out this story, we’ve received unconfirmed reports, and I emphasize ‘unconfirmed,’ that the same reservations are being expressed by law enforcement and emergency service personnel.

Anchor Dani: “Barbara, will there by enough people to man these critical positions?”

Barbara Parsons: “I don’t know Dani, no one will speak on the record, I guess we will have to wait for 3:00 p.m. to see just who reports for work and who doesn’t.

Dani, not everyone is hesitant to go on camera. Janet Dans is a laboratory technician at the (medical) Center.

- *Cut to a video clip of Janet Dans in scrubs.*
- *Super:* **Janet Dans–Lab Technician**

Janet Dans: “Our supervisors are telling us that there is no danger of contracting Rift Valley fever in the lab as long as we take standard barrier precautions like those we use in dealing with material that may be infected with the AIDS virus.

“But if that’s so, why do technicians working in government labs with the RVF virus, all get vaccinated before they ever work with the virus?”

- *Cut to Anchor Dani*
- *Graphic over her shoulder shows ambulance/emergency graphic.*

Anchor Dani: “Janet has asked the question that is on the minds of a lot of the healthcare and emergency workers here in (City). But that hasn’t stopped a flood of people making their way to area emergency treatment facilities, most of them complaining of various symptoms.. all afraid they’ve got it.

We go to News 11 reporter, Ted Brady.”

- *Cut to video of Ted Brady with microphone standing near emergency room entrance.*

Ted Brady: “Joni Whitman watched the press conference from (State capital) this morning, then went to care for her son Earl, Jr., age 16 months, who had just awakened.

- *Cut to video of Joni holding Earl, Jr.*

Joni Whitman: When I went to get Earl, I saw that his little arms was just eat up with bites, so I brought him here. We’ve been here for hours and haven’t seen no one yet. There’s just so many sick people here.

- *Back to Ted Brady on camera.*

Correspondent Brady: “That’s pretty much the situation at every emergency room in town, Dani. Many people are worried about RVF.”

- *Fade to black.*

Number 14—Week 1, Wednesday, 4:15 p.m.

- *Open on black.*
- *Super the date and time: “Wednesday, 4:15 p.m.”*
- *Fade in to WNN Headline News with* **“Crisis in America”** *across the bottom of the screen.*

WNN Anchor: “ Help has arrived in the embattled (City) area of Northwest (State) now in the clutches of Rift Valley fever, the first such outbreak in the U.S. Advance units of the (State) National Guard, arrived this afternoon in (City), ordered there by (State) Governor (name) to join the front lines of a beefed-up program of mosquito eradication. Guard personnel were quickly outfitted for the task and made a part of efforts to control the outbreak.

“So far, 150 cases of the deadly disease have been confirmed with 6 to 8 deaths attributed to the viral infection, which until now has been found mostly in Africa.”

- *While Anchor is delivering lines, show video of humvees pulling up, guardsmen and women being shown equipment, then being loaded in trucks to go out for mosquito control.*
- *Fade to black.*

Number 15—Week 1, Wednesday, UBC Evening News

- *Fade in to black.*
- *Super Wednesday, UBC Evening News.*
- *Fade in to national newscast.*

Anchor James Guest: "The nation and especially residents of the area around (City) are trying to come to grips with an outbreak of an exotic viral infection that devastates certain species of farm animals and attacks humans as well.

"The outbreak of Rift Valley fever that has spread through (City), (State), has wiped out animal populations on many area farms. The number of cases of human infestation has climbed past 150 with 8, possibly 9 deaths attributed to RVF.

"Law enforcement agencies, including the FBI, are still trying to determine whether or not the introduction of the disease into (State) was the work of terrorists or a natural occurrence.

"Dr. A.D. Hendrix of the Johns Hopkins Bloomberg School of Public Health spoke with Correspondent Ron Crosby earlier today."

Ron Crosby: "Dr. Hendrix, are we helpless when faced with this new viral infection, Rift Valley fever?"

Dr. A. D. Hendrix: "No, Ron, we are not helpless in regards to Rift Valley fever. Granted, it's an exotic virus that is unknown to most people in this country. But the Centers for Disease Control and Prevention and the U.S. Army Medical Research Institute of Infectious Diseases have been studying and treating the disease in Africa for years. We know that an aggressive and thorough mosquito eradication program will halt the spread of the disease in (State), so we're not helpless."

Ron Crosby: "But what about those who have contracted the disease?"

Dr. A. D. Hendrix: "Most people who contract RVF respond well to supportive therapies. There aren't any specific therapies available yet, although ribavirin, which is a commonly available anti-viral drug, has been shown to have some value as a treatment for RVF in laboratory animals."

1. *Cut to video of lab with workers, ghost out and super information on this background. Ron Crosby reads the points as they are put up.*

Ron Crosby: "After our discussion of Rift Valley fever with Dr. Hendrix, I researched the connection of Rift Valley fever and ribavirin. A MEDLINE computer search of the National Library of Medicine turned up no less than 19 references in published medical journals dealing with the use of ribavirin in the treatment of Rift Valley fever in laboratory animals. There were no references detailing the use of ribavirin to treat RVF in humans.

"But the story does not end there. When I contacted the U.S. Army Medical Research Institute of Infectious Diseases, which is called USAMRIID, they refused comment. But, in an off-the-record comment, a scientist at USAMRIID told me USAMRIID's own doctors have used ribavirin in other countries to treat a small number of humans infected with RVF."

- *Cut back to anchor.*

Anchor James Guest: "We are joined now by Lt. Colonel Tom Paine of the Public Affairs Office of the U.S. Army Medical Research Institute of Infectious Diseases, at Ft. Detrick, Maryland. What about the use of ribavirin to treat humans, Colonel?"

- *Colonel Paine is on location in Maryland.*

Colonel Tom Paine: “Ribavirin is simply not a proven or established treatment for Riff Valley fever in humans. The studies that your correspondent cited concerned laboratory animals, not humans. When ribavirin has been used in animals, it has always been in the form of an intravenous injection, not in a capsule or in the inhaled form, the two forms of the drug that would be available to humans. None of the evidence that’s available supports ribavirin as the ‘magic bullet’ for this current outbreak in Northwest (State).”

- *Fade to black.*

Number 16—Week 1, Wednesday, WNN Late Night News

- *Fade in to black.*
- *Super: Wednesday, WNN Late night News*
- *Fade in to national newscast with WNN typesetting, super* **RVF** *slug over his shoulder*

WNN Anchor: “News that the anti-viral drug, ribavirin, has value as a treatment for Rift Valley fever, has created a growing demand for the drug in (City), (State), scene of the outbreak. Physicians and some registered nurses report that they have been contacted even at home by patients with requests for prescriptions for the drug, in spite of the fact the ribavirin’s value as a RVF treatment, has been debunked by government health authorities.

- *Graphic changes to* “**Robbery”** *graphic.*

“At least two (City) residents who were anxious to obtain ribavirin, took the unconventional route of holding up pharmacies. Said one of the pharmacy victims, ‘They didn’t want my cash, or my narcotics, they just wanted the ribavirin.’”

- *Fade to black.*

Number 16A—Week 2, Thursday, Morning, *The Times of (City)*

- *Fade in on black background*
- *Super the date: Thursday, Morning*
- *Dissolve to ghosted out newspaper page background on which bullet points will be supered.*
- *Audio narration is delivered before headline and bullet points from the story are supered.*

Off-camera audio: “In a joint statement issued last night on Wednesday, the Department of Health and Human Services, the Centers for Disease Control, the U.S. Department of Agriculture and the Environmental Protection Agency commented on the aggressive mosquito control efforts now underway in the (City) area. In a *Times of (City)* article appearing in its Thursday edition, the following points were made”:

The spread of Rift Valley fever can be controlled by mosquito elimination efforts, just as other mosquito-borne diseases can be controlled, such as St. Louis encephalitis, Easter Equine encephalitis, West Nile fever, and malaria.

CDC and other public health officials recognize that some questions about Rift Valley fever and its treatment remain unanswered, although they have been studying this illness in those parts of the world where it occurs routinely.

The disease spread can be stopped in both humans and animals through the use of EPA-approved larvacides and adulticides in mosquito-control efforts. Local health officials will provide details about their use of these EPA-approved agents and the schedules for their application.

During this early stage of the disease outbreak, federal health officials offer the following suggestions:

- avoid outdoor activities during the early morning and evening hours when mosquitoes are most likely to bite
- when outdoors wear insect repellent that contains DEET
- wear long-sleeved shirts and pants when outdoors
- eliminate all standing water on and around your property, including pet water bowls, flower pots, birdbaths and stagnant pools.

Anyone who has flu-like symptoms including fever, generalized weakness, back pain, dizziness and extreme weight loss at the onset of illness should consult a healthcare provider. Supportive therapy is the appropriate treatment for this illness. Health officials will continue to explore all possible treatment therapies to help patients with this infection.

Healthcare professionals should work with public health officials to identify cases among humans and should consult with them regarding current treatment guidelines. USDA asks that veterinarians consult with state and local veterinary health officials regarding disease control among livestock. Although it is reasonable to be concerned about the introduction of a new disease into the Western Hemisphere, CDC reassures the public that, based on current knowledge, it appears that most persons who acquire the infection do not exhibit symptoms and will not require medical care. To learn more about Rift Valley fever, go to the CDC Web site at www.cdc.gov

- Fade to black.

Number 17—Week 2, Thursday, 10 a.m. Local television news

- *Fade in from black.*
- *Super: Thursday, 10:00 a.m. story in continuing local coverage.*
- *Setting is local newsroom.*
- *Graphic is* “**Outbreak in America”.**

Anchor Dani: “First it was the disease itself—watching a loved one die of Rift Valley fever or standing by helplessly day by day as a family member slips away closer and closer to death. But, as of this morning, the families of RVF victims close to death have an added burden to deal with.

- *Graphic changes to* “**Forced Cremation**?”

“News 11 has learned that this morning the (City) Morticians Association told the mayor of (City) that members of the Association will not offer funeral services to RVF victims unless their bodies have been cremated.

“Opal Krans’ husband, Victor, died of RVF.”

- *Cut to video close-up of Opal Krans*

Opal Krans: “I’m sure just like a lot of people…as my husband and I matured, it just made good sense to plan ahead….You know, we gave a lot of thought to funeral arrangements and such—not just for our sakes, but for the kids….in large part, I think, just so the other wouldn’t have to deal

with it at a time of emotional …We planned ahead. We bought plots…My goodness, they've been paid for years. And now …We both had strong feeling against cremation. It's not what Victor wanted—it's not what I want. And now to be told, I don't even have a choice…It's not right, it's too much …it's just too much."

- *Cut back to Anchor in studio*

Anchor Dani: "Spokesman for the Mayor's office told News 11 that they have received a number of phone calls from RVF victims' families protesting this new policy because it violates their religious beliefs or the wishes of their relatives.

"News 11 will stay on top of this story for you and report any new developments."

- *Fade to black.*

Number 18—Week 2, Thursday, WNN Cable News at noon

- *Fade in from black.*
- *Super: Thursday, Cable News at Noon.*
- *WWN Anchor at standard news desk doing "back and forth" with a reporter in (City). Reporter is standing in front of City Hall.*
- *Graphic over his shoulder says,* **"Rift Valley fever Emergency".**

WNN News Anchor Fred: "A government decision made this morning to aid the on-going struggle in (City), (State), against an outbreak of Rift Valley fever has the potential to create controversy.

"For more we go live to (City), (State), and WNN correspondent, Jennifer Adams.

"Jennifer—"

- *Split the screen so that Anchor and Correspondent have equal prominence*. *Jennifer is standing in front of (City) skyline.*

Jennifer Adams: "In a conference call with the mayor of (City), (State), (State) state epidemiologist and local medical leaders, officials of the Centers for Disease Control and the U.S. Army Medical Research Institute of Infectious Diseases announced that the White House is studying the possibility of directing the Army to release 2,000 doses of its Rift Valley fever vaccine for use in the (City) metropolitan area."

Anchor Fred: "Are 2,000 doses enough, Jennifer? (City) is a large area, isn't it?"

Jennifer Adams: "Fred, this vaccine has been used primarily for the protection of laboratory workers who study RVF virus. The Army says they have more than 2,000 doses, but the other lots they have need to be potency-tested, which takes a long time."

Anchor Fred: "How long, Jennifer?"

Jennifer Adams: "No one is saying how long a 'long time' is, Fred, but in any case before any vaccine is released there are other questions to be answered."

Anchor Fred: "Like what?"

Jennifer Adams: ”One issue is how the vaccine will be used. It’s classified by the Food and Drug Administration as an Investigational New Drug and it’s authorized for use only under a highly specific protocol. But because the drug may be used in this (State) emergency, the FDA is developing a more permissive protocol that would enable the vaccine to be used under less stringent regulations. There are also questions about the dosing schedule and also who is going to receive the vaccine. Some vaccine experts suggest that lab workers who work with potentially infected blood and tissue samples, should get the vaccine first and that veterinarians should also get priority.”

Anchor Fred: “Who else?”

Jennifer Adams: “Not just who else, Fred. What else? is appropriate because another group of scientists say the outbreak can best be controlled by using the vaccine on animals, not people.”

Anchor Fred: “So when are the issues going to be decided?”

Jennifer Adams: “That’s a good question, Fred. The federal authorities told the mayor that they hope to reach consensus on these issues soon and they will keep the mayor apprised of their progress.

“But the possibility of vaccine being available is definitely on the table now.

“There have even been some incorrect news reports which said the vaccine was being shipped,...that is completely wrong.”

Anchor Fred: “We didn’t say that. WNN wouldn’t carry that kind of report. Thanks, Jennifer. You’ll stay on top of this vaccine story for us won’t you?”

Jennifer Adams: “I’ll stay with it, Fred.”

- *Fade to black.*

Number 19—Week 2, Thursday, WNN Cable News at 3:00 p.m.

- *Fade up from black.*
- *Super-Thursday, 3:00 p.m. WNN Cable Newscast*
- *Fade to Anchor at news desk.*

Anchor Fred: “(City), (State), Northwest (State) city that is the scene of the first Rift Valley fever cases in the U.S. has another budding problem.

WNN Correspondent Jennifer Adams has the story.”

- (*Cut to close-up (name), Fire Chief of the (City) Fire Department.)*
- *As he talks, Jennifer Adams does voice-over.*

“This morning’s revelation that federal authorities are considering release of 2,000 doses of a special Rift Valley fever vaccine for use in (City) caused groups that might get the vaccine to immediately make their cases.”

- *Video continues, but with Chief (name’s) voice.*

- *Super:* **“(Name) – Fire Chief, (City) Fire Department**”

Chief (name): “Our firefighters and EMS people are the front lines in this war and we think that it just makes good sense to vaccinate those on the front lines and their families if you want to keep us in the fight.”

Jennifer Adams: “Are you saying that if your firefighters and EMS people and their families don’t receive the vaccine, you won’t stay on the job?”

Chief (name): “No! I’m not saying that at all! I am saying that if one of us goes down with the fever, who is going to step up and take their place? ”

- *Cut to video of Emergency Room nurse with voice-over of Jennifer Adams*
- *Super*: **“Marge Janis, ER Nurse**”

Jennifer Adams: “Marge Janis is an ER nurse at (name) Medical Center.”

- *Video continues with Marge Janis doing the audio.*

Marge Janis: “This place has been a madhouse since RVF was identified. Everyone that comes in thinks they have it and some do. I’m a professional and I take every precaution, but I’d still like to have the vaccine as a hedge against an accident or the unexpected.”

- *Cut to video of lab workers with Jennifer Adams doing voice-over.*

Jennifer Adams: “Lab personnel tell us they deserve the vaccine because they work with tissue and blood that may be infected. They also tell us that government lab workers are vaccinated against RVF before they ever work with the virus. Floor nurses are making their case and even administrative office workers, who have handled the paperwork of RVF victims, also feel they deserve the vaccine. If the vaccine is made available, there will be no shortage of people ready to receive it.

“This is Jennifer Adams, WNN News in (City), (State).”

- *Fade to black.*

Number 20—Week 2, Thursday, WNN Cable News—Late Night

- *Fade in to black.*
- *Super-Thursday, Late Night WNN Cable News*
- *Fade in on the WNN Anchor with graphic over his shoulder.*

Anchor Fred: “This just in. The Office of Homeland Security in Washington, DC, has just announced that the outbreak of Rift Valley fever in (City), (State), is an act of bioterrorism, not a natural occurrence.

“A spokesman for the Office of Homeland Security Director Tom Ridge said: ‘The pattern of infection makes it almost certain that the introduction of Rift Valley fever virus was an intentional act. All resources of the FBI and other law enforcement and intelligence agencies are being brought to bear on identifying and apprehending the responsible parties.’”

Anchor Fred: “I repeat, the Office of Homeland Security has just announced that the infection of (City) in Northwest (State) with Rift Valley fever was an act of bioterrorism.”

- *Fade to black.*

Number 21—Week 2, Friday, Local newscast-7:00 a.m., (x)DT

- Fade up from black.
- Super - Friday, Local News, 7:00 a.m. (x) DT continuing coverage
- Fade in to newscaster at desk.
- *Anchor Dani is seated at the regular news desk with the cityscape background.*
- *Super over Anchor*—**News 11—Dani Cates**

Anchor Dani: “Good morning and welcome to our continuing coverage of ‘Outbreak in America,’ the Rift Valley fever outbreak here in (City). We will now join the Honorable (name), mayor of (City), in his offices at (name) in downtown (City).

“Good morning, Mayor. We know how busy you’ve been these last few days and we appreciate you taking the time to be with us.”

- *When anchor Dani mentions being joined by the mayor, we go to a split shot showing both the anchor and Mayor (name). When the anchor completes asking the first question, and the mayor starts to speak, we go full screen on the mayor.*

Mayor (name): “Good morning, Dani. I appreciate News 11 making this time available to me.”

Anchor Dani: “Is there anything new you can tell us about the RVF outbreak?”

Mayor (name): “Dani, I have just completed a conference call that included officials from the (State) Department of Health, from the U. S. Centers for Disease Control and from the U. S. Department of Agriculture. After hearing what these experts had to say, I believe that we’re on the right track to control and eradicate this disease.

“First, a word about those who have contracted RVF. Diagnostic tests have not been completed on the latest hospital admissions, but it looks like we have 165 to 168 confirmed cases with 10 fatalities. We’re hoping to see the number of new cases begin to decrease rapidly.

“This morning, the U.S. Department of Agriculture took another step to halt the spread of RVF, when they restricted the movement of cattle in North (State) and parts of (neighboring states). This is a quarantine, Dani, and I have been assured by CDC and the Department of Agriculture that this quarantine of cattle, along with aggressive mosquito control is what’s needed to halt the spread of RVF to other areas. There have been a number of RVF outbreaks in other parts of the world and when aggressive mosquito eradication was undertaken along with animal quarantine, the spread of the disease was stopped. I know that’s what the people of (City) want. Yesterday, our mosquito control efforts were helped by the arrival of units of the (State) National Guard. They were quickly put to work as part of our mosquito spraying program.”

When the mayor completes his answer, the split screen with the anchor is re-established so the anchor can ask her final question.

Anchor Dani: “Mayor, have you been given any information on the terrorists who are responsible for this?”

Mayor (name): “I have no information on who did this or how it was accomplished, Dani. But, I am confident that the people responsible for this

act of terrorism will be identified and punished.

“Dani, it just seems like our enemies continue to mistake the peaceful nature of our society for weakness. As Americans, we know that’s not the case. These people will be punished, I’m confident of that, but right now, here in (City), we’ve got a job to do and that’s to bring this outbreak under control and care for those who have become sick and those who have lost loved ones. That’s what we’re called on to do right now, and I’m proud of the way our people are handling this situation. That’s the best way to respond to the terrorists, to show them that we will not be broken by their actions.”

- *Fade to black.*

Number 22—Week 2, Friday, WNN News, Financial Report—9 a.m. (X)DT

- *Fade up from black.*
- *Super-Friday, WNN Cable Financial News Report at 9 a.m.*
- *Fade in to Financial Anchor Phillip Vest at news desk with graphics over his shoulder.*

Financial Anchor: “The outbreak of Rift Valley fever in Northwest (State), which has now been confirmed as an act of bioterrorism, has hit several (State) industries with withering blows.

“Restaurants throughout (State) report that customers are avoiding all beef and pork products and are only ordering chicken, fish, or seafood. The move away from beef and pork is so complete, some restaurant owners fear losses on stores of meat they already have on hand.”

- *As the Anchor finishes the copy above, video cuts to a “two” shot with the Anchor sitting next to “Saudi” diplomat in business suit. Both are still seated at the anchor desk.*

Financial Anchor: “I am joined now by Addulah Al-Hakim, who is a diplomat with the embassy of the Kingdom of Saudi Arabia. Mr. Al-Hakim, thank you for joining us. In the year 2000, your country experienced an outbreak of Rift Valley fever along your border with Yemen, is that correct?”

Al-Hakim: “Yes, that is correct. It was very difficult to eradicate because that border region is inhabited by nomads who are extremely independent. It was difficult to control their movements to see that the actions necessary to stamp out the virus were taken.”

Financial Anchor: “Mr. Al-Hakim, you’ve just heard the story about diners avoiding meat products in (State) restaurants. Have you any comments?”

Al-Hakim: “The same thing happened in my country during the outbreak in 2000. Regardless of what we told them, people would not eat meat.”

- *Fade to black.*

Number 23—Week 2, Friday, WNN Cable News, Financial Report—9:15 a.m., (X)DT

- *Fade in from black.*
- *Super-Friday, 9:15 a.m. WNN Cable Financial News*
- *Fade in to Anchor sitting at desk-as he speaks, graphic appears over his shoulder.*

Financial Anchor: “The Chicago Board of Trade opened sharply down in all sectors. As expected by many analysts, turmoil has been created by the widening scope of the Rift Valley fever crisis in Northwest (State). Confirmation that the outbreak is an act of bioterrorism gave many investors the jitters and this morning’s announcement from the Department of Agriculture of an animal quarantine in (State), (neighboring states), was all that it took to start the downward spiral. Corn, soybeans, wheat, and cattle futures and pork bellies were all driven lower right from the opening bell.

“Market analysts are at a loss to predict just where we are headed since we have never before experienced such a crisis.”

- *Fade to black.*

Number 24—Week 2, Friday, WNN Cable News, Financial Report —9:58 a.m., (X)DT

- *Fade up from black.*
- *Super-Friday, 9:58 a.m. - WNN Cable Financial News Network.*
- *Fade up to Financial Anchor with graphic over his shoulder.*

Financial Anchor: “Traders are taking a hit on the New York Stock Exchange. After only 1 hour and 28 minutes of trading the Dow Jones Industrial Average was down 247.5 points or 2.51%. All sectors of the market have shared in losses felt from the opening bell - agriculture, tourism and gambling stocks, especially those of companies operating in (region) states have borne the brunt of the onslaught.”

- *Fade to black.*

Number 25—Week 2, Friday, WNN Cable News, Financial Report—10:15 a.m., (X)DT

- *Fade up from black.*
- *Super-Friday, 10:15 a.m., WNN Cable Financial News Network.*
- *Fade up to Financial Anchor with graphic on his shoulder.*

Financial Anchor: “The Centers for Disease Control and Prevention has just issued a statement in connection with the terrorist-initiated Rift Valley fever infestation in Northwest (State). In addition to discussing how the RVF virus is transmitted and detailing precautions that can be taken to avoid the mosquitoes that transmit the virus, the statement seeks to dispel fears the public might have about the nation’s meat supply. These fears are reflected in the report that (State) restaurant patrons are foregoing beef and pork in favor of poultry, fish and seafood.

“For the full text of the statement, here is WNN correspondent Reid Watley.”

- *Cut to Reid Watley in studio with graphic over his shoulder. As he intros the statement, the camera widens and repositions so that the statement can be supered to his side.*

Reid Watley: “In an attempt to set the record straight and dispel fears about contracting Rift Valley fever by eating meat, the Centers for Disease Control and Prevention has just issued this statement”:

‘In developing areas of the world where RVF is endemic (areas where the virus is present), animal herdsmen, abattoir workers, and other individuals who work with animals have an increased risk for infection. Persons in high-risk professions, such as veterinarians and

slaughterhouse workers, have an increased chance of contracting the virus from an infected animal. Avoiding exposure to blood or tissues of animals that may potentially be infected is an important protective measure for persons working with animals in RVF-endemic areas.

Current research indicates that humans can get RVF as a result of bites from mosquitoes and possibly other blood-sucking insects that serve as vectors. Humans can also get the disease if they are exposed to either the blood or other body fluids of infected animals. This exposure can result from the slaughtering or handling of infected animals or by touching contaminated meat during the preparation of food. Infection through aerosol transmission of RVF virus has resulted from contact with laboratory specimens containing the virus.

Understandably, some persons may not be comfortable preparing meat at this time; however, meat processed in the United States must go through rigorous inspection and handling steps which would eliminate all but a theoretical risk of contracting RVF from meat. At this time, CDC does not recommend persons avoid eating meat bought and prepared in the United States.

CDC currently recommends the following actions by the public to avoid RVF:

• Avoid outdoor activities during the early morning and evening hours when mosquitoes are most likely to bite;

- **Wear insect repellent that contains DEET when outdoors;**
- **Wear long-sleeved shirts and pants when outdoors. Eliminate all standing water on and around your property if possible, including pet water bowls, flower pots, birdbaths, and stagnant pools.**

Persons who have flu-like symptoms, including fever, generalized weakness, back pain, dizziness, and extreme weight loss at the onset of illness should consult with their health care provider. Supportive therapy is the appropriate treatment for this disease. Health officials will continue to explore all possible treatment therapies to help patients who have been infected with this disease.

CDC recommends health care professionals work with public health officials to identify cases among humans and consult with them regarding current treatment guidelines. The USDA asks that veterinarians also consult with state and local veterinary health officials regarding disease control among livestock.

Current knowledge regarding this disease is that most persons who become infected do not develop symptoms and will not require medical care.

For more information about Rift Valley fever, go to www.cdc.gov '"

- *As Watley reads the statement, it crawls up the screen next to him.*
- *Statement is supered on screen.*
- *Fade to black.*

Number 26—Week 2, Friday, WNN Cable News —10:45 a.m.

- *Fade up from black.*
- *Super-Friday, 10:45 a.m. WNN Cable News*
- *Fade into WNN Anchor at desk.*

Anchor Fred: "In the latest concerning the rapidly developing situation concerning the outbreak of Rift Valley fever in Northwest (State), the

governors of the adjoining states of (names) have added their voices to those who are asking for stronger measures to prevent the spread of RVF to other areas.

“This morning, the U.S. Department of Agriculture announced the immediate curtailment of the movement of cattle and other farm animals in the affected areas of (names of all states involved).

“In their statements, issued simultaneously by press secretaries of both governors, they said,

‘**Since no developed country has ever been exposed to Rift Valley fever, there is no model to prove that mosquito control and animal quarantine will be sufficient to halt the spread of the infection. We strongly urge the Federal Government to take every step possible to prevent the further spread of this disease.’”**

“For more on this, we are joined by Dr. Bob Arness, WNN’s Chief Medical Correspondent.”

- *Go to a two-shot of the Anchor and Dr. Bob Arness.*

Anchor Fred: “Bob, the governors’ statement stopped short of calling for a total quarantine of the area, including humans. Do you think that’s next?”

Dr. Bob Arness: “That’s a big step, but what else can be done? Don’t forget Fred, that for a time infected humans have enough virus in their blood to serve as a reservoir of virus for mosquitoes. If they are bitten by a mosquito during this time, that mosquito could transmit the infection to other people.”

Anchor Fred: “Let me get this right. If one of these human reservoirs, as you called them, travels into an un-infected area and is bitten by a mosquito, the virus has been spread?”

Dr. Bob Arness: “As long as he is bitten by the right type of mosquito, and that mosquito then bites a suitable victim.”

- *Fade to black.*

Number 27—Week 2, Friday, WNN Cable News—11:00 a.m., (X)DT

- *Fade up from black.*
- *Super-Friday, 11:00 a.m. WNN Cable News.*
- *Fade into WNN news desk with Anchor at desk and graphic over his shoulder.*

Anchor Fred: “(State) Governor (name) has quickly weighed in on this morning’s developments concerning the Rift Valley fever crisis in Northwest (State).

“The U.S. Department of Agriculture announced this morning the immediate curtailment of the movement of cattle and other farm animals in the affected area and also in parts of the adjoining states of (state names).

“Later in the morning, in a joint statement, the governors of (names of adjoining states) called on the federal government to take even stronger steps to interrupt the spread of the disease.

"Speaking through his press aide, (name of governor of state primarily affected) seemed to dismiss mounting calls for a total quarantine of the affected area as he said:

'Quarantining farm animals and aggressively pursuing mosquito control measures make up the best course of action at this time.'"

Anchor Fred: "The calls for a total quarantine seem to be strongest from people that are close to the affected area. For more we go to WNN correspondent, Jennifer Adams. Jennifer ...."

- *Cut to video of Jennifer Adams in rural setting.*

Jennifer Adams: "Prentice Swank lives in (name of another town in primarily affected state), south of (City of primary infection) and the infestation of Rift Valley fever and he and his neighbors are worried."

- *Close-up of Prentice Swank.*

Prentice Swank: "Last night me and my neighbors here in (Town) met with some fellas from (names of adjoining towns) to talk about this thing and this morning I got the president of the (county government agency) on the phone."

Jennifer Adams: "What was the nature of your conversation?"

Prentice Swank: "I told him that folks around here wanted some action! I told him that Washington ain't going to help us—that we needed to take action ourselves."

Jennifer Adams: "What kind of action?"

Prentice Swank: "We need to slaughter all the cattle close to (City) that might have the stuff. Everybody coming out of the area needs to be stopped and the fire department needs to hose down everybody coming out of (City) with disinfectant. Keep that stuff from spreading."

Jennifer Adams: "The CDC says that the virus is spread by mosquitoes and that mosquito control is the answer."

Prentice Swank: "Well, ma'am, the CDC don't live in (name) County, so it's no skin off their noses if they're wrong. We don't need to depend on the government. Why, anybody coming out of the city may have this stuff and spread it all around. We need to take action and bottle this stuff up now."

Jennifer Adams: "Fred,...there is clearly a body of thought developing that eradicating mosquitoes and controlling the movement of farm animals may not be enough."

Anchor Fred: "Jennifer is it your sense that this belief that humans can spread the disease is gaining momentum?"

Jennifer Adams: "Yes, Fred, the fear that the disease can be spread by humans is very real. My camera crew and I came out of (City) this morning to do this interview and when people found out we had been in (City), they avoided us—they didn't say it, but it was apparent that they were concerned that we might be RVF carriers. We tried to talk to 16 people before Prentice Swank agreed to talk to us."

Anchor Fred: “Well, Jennifer, what’s the answer?”

Jennifer Adams: “Fred, I’m not sure I even know the question.”

- *Fade* to black.

Number 28—Week 2, Friday, Local Evening News—6:00 p.m., (X)DT

- *Fade in from black.*
- *Super-Friday, Local news at 6 p.m.*
- *Fade to local Anchor.*

Anchor Dani Cates: “The toll from the bioterrorism-induced infestation of Rift Valley fever which still grips (City) grows each day. 165 to 168 confirmed cases of RVF are being treated at area medical facilities with the number of deaths attributed to the disease now at ten.

“There is another toll being exacted by the outbreak: the isolation of the city of (City).

“Health Reporter Barbara Parsons has more.”

- *A series of video clips is run with appropriate voice-over from Barbara Parsons.*

Barbara Parsons: “The isolation of (City) might have been started by a U. S. Department of Agriculture order issued this morning, which curtailed the movement of cattle and other farm animals in our area and parts of (names of neighboring states).

That order was followed by a joint statement from the governors of (neighboring states) that seemed to hint at the need for a human quarantine.

“As the day went on it became apparent that government action wasn’t necessary to isolate (City).

“At noon, tomorrow morning’s baseball game between (name) High School and (City) College Prep was abruptly canceled by the (name) school principal, with the explanation: ‘We’ll talk about rescheduling when this fever thing is over.’

“The (name) High School wrestling teams’ invitation to a weekend tournament in (name) was withdrawn at 2 p.m. No explanation for rescinding the invitation was given to the (City) high school.

“A flood of cancellations at every (place of business) has dropped expected occupancy for the weekend to less than 25%.

“And the old adage about ‘flying the friendly skies’ didn’t hold true either when American Airlines canceled its flights into the (City) Airport. Airline officials gave the explanation that mosquitoes carrying the virus might board its planes while they were on the ground here.

“And finally, those huge truck stops perched on every road and Interstate Highway running through our area are really hurting. Some report gasoline sales down 60 to 70 percent. One manager said, ‘I know people haven’t stopped traveling—they’re just not stopping here.’”

- *Fade to black.*
