# Supplementary material for: Community Reaction to Bioterrorism: Prospective Study of Simulated Outbreak
Source: Emerg Infect Dis. 2003 Jun;9(6):708–12. doi: 10.3201/eid0906.020769 (PMC3001393; doi:10.3201/eid0906.020769)
Supplement: Appendix 2 — Note: The following questionnaire is from an outbreak simulation. These events did not occur. [file 02-0769-app2-s2.pdf]

Appendix 2

Note: The following questionnaire is from an outbreak simulation. **These events did not occur.**

Medical First-Responder Group: Session 1

First responders are making an increasing number of calls to treat sick people and transport them to area hospitals. In the hospital emergency rooms, nurses and others talk about the increasing number of patients they are seeing, some of whom are quite sick and are likely to die. No one seems to know what the disease is. You ask your supervisors what’s going on, what the disease outbreak is all about, how dangerous it is to you and your family, and what you can do to protect yourself and your family. Your supervisors reply that they don’t know anything more than anyone else, including doctors. It may just be some kind of flu, or something else, and people are trying to find out what’s up, they say, but, at the moment, no one seems to know much about “this problem.” Your supervisors say the experts are convinced it’s not anthrax or smallpox, and, they say, it may be naturally occurring and not bioterrorism. Your supervisors say they are convinced the doctors and public health experts are being honest and not hiding anything from them. Your supervisors remind you to use standard, universal precautions whenever you come in contact with a patient, and always wear gloves. In addition, “just to be on the safe side,” your supervisors tell you to wear a surgical mask, as well. When someone asks about taking antibiotics, your supervisors reply that the doctors have told them that, until they know what the disease is, they can’t know what antibiotics or other treatment to prescribe.

1. What are you likely to do regarding your job? (Choose the ONE answer that is closest to your decision.)

(A) Stay on the job and take no precautions other than wearing gloves/mask and taking universal, standard precautions.

(B) Stay on the job, follow standard precautions (including wearing mask and gloves), and try to get some kind of antibiotics from my local doctor or someone else.

(C) Try to take some sort of job action (by myself or with others) in order to cut down on the number of calls I have to make.

(D) Stay away from work by taking sick leave or annual leave.

(E) Take leave without pay.

(F) Quit.

(G) Other (explain briefly).

2. In your own words, briefly describe the kind of information or assurances you would want someone to give you that would help convince you to stay on the job at this time.

3. In your own words, briefly identify who should provide you with that information or those assurances (mentioned in the last question).

4. What role would your spouse/significant other have in helping you make your decision about which choices (see question one, above) you would make regarding your job? (Choose the ONE best answer.)

(A) My spouse/significant other and I would have to agree on the choice; I would take no action regarding my job that my spouse/significant other did not agree with.

(B) My spouse/significant other and I would not have to agree on the choice; I would be willing to take action regarding my job that my spouse/significant other did not agree with.

(C) Not applicable; I don't have a spouse or significant other.

(D) Other (explain briefly)

5. At this point, what one source of health information will have the greatest influence on your decision regarding your job? (Choose ONE answer that represents the source of greatest influence.)

(A) My coworkers.

(B) My supervisor.

(C) My union representative.

(D) My own doctor.

(E) Friends and neighbors.

(F) Friends/loved ones who know something about medicine.

(G) Local public health officials.

(H) Local politicians or other non-medical local officials.

(I) Newspapers, television, radio reports.

(J) My loved ones, regardless of whether or not they know anything about medicine.

(K) Other (identify and describe briefly)

6. What actions are you likely to take about your family/loved ones? (Choose the ONE answer that is closest to your decision.)

(A) Nothing unusual.

(B) Try to restrict their contacts with other people; encourage them to stay more at home, perhaps even keep my kids from school..

(C) Try to get some kind of antibiotics for them and me.

(D) Try to restrict their contacts with other people; encourage them to stay more at home, perhaps even keep my kids from school AND try to

get antibiotics for them.

(E) Send my family out of town, to visit friends or relatives elsewhere, but I'll stay home and on the job.

(F) My spouse/significant other can stay here and stay on the job, but I'll leave with my family, and we'll go to visit friends or relatives elsewhere.

(G) Leave with my family, and we'll ALL go to visit friends or relatives elsewhere.

(H) I don't have family or loved ones here, but I would stay in town.

(I) I don't have family or loved ones here, but I would leave town.

(J) Other (explain briefly)

Medical First-Responder Group: Session 2

1. Based on the news coverage you've just heard and watched, which one of the following statements about the transmissibility of Rift Valley fever do you most agree with? (Choose ONE.)

(A) There is little or no possibility that I can catch Rift Valley fever from another person.

(B) There is a moderate risk that I can catch Rift Valley fever from another person.

(C) There is considerable risk that I can catch Rift Valley fever from another person.

(D) I don't know what the risk is that I can catch Rift Valley fever from another person.

(E) Other (explain briefly)

2. Based on the news coverage you've just heard and watched, which one of the following statements about the quality of the Federal Government's information do you most agree with? (Choose ONE.)

(A) The Federal experts from the CDC and the Department of Agriculture told me all I needed to know about Rift Valley fever, and the other experts that followed them added nothing of importance to my understanding of this disease.

(B) The Federal experts from the CDC and the Department of Agriculture told me much of what I needed to know about Rift Valley fever and the other experts that follow them contributed additional worthwhile information that helped me to understand this disease better.

(C) I didn't think the Federal experts from the CDC and the Department of agriculture were entirely open and honest, and the other experts that followed them raised important points.

(D) Other (explain briefly)

3. Based on the news coverage you've just heard and watched, which one of the following statements about the importance of additional information from your local public health authorities do you most agree with? (Choose ONE.)

(A) After hearing the Federal experts, I do not need to hear local public health officials talk about this disease to understand its importance to my family and me.

(B) After hearing the Federal experts, I would like to hear local public health officials talk about this disease to understand its importance to my family and me.

(C) After hearing the Federal experts, I still need to hear local public health officials talk about this disease to understand its importance to my family and me.

(D) Other (explain briefly

4. Where do you think the press conference you just saw should have been held? (Choose ONE.)

(A) In my city—where the epidemic is

(B) The state capital

(C) Washington, DC

Medical First-Responder Group: Session 3

1. Would you try to get your doctor or your pharmacist to provide you with ribavirin for yourself? (Choose ONE.)

(A) Yes.

(B) No.

(C) Undecided.

2. Would you try to get your doctor or your pharmacist to provide you with ribavirin for your family/loved ones? (Choose ONE.)

(A) Yes.

(B) No.

(C) Not applicable to me; I don't have family/loved ones here.

(D) Undecided.

Medical First-Responder Group: Session 4

1. If the vaccine becomes available, would you ask to get it for yourself? (Choose ONE.)

(A) Yes.

(B) No.

C) Undecided.

2. If the vaccine becomes available, would you ask to get it for your loved ones/family? (Choose ONE.)

(A) Yes.

(B) No.

(C) Not applicable to me; I don’t have family/loved ones here.

(D) Undecided.

3. If the vaccine becomes available but is in short supply, would you demand that you get it for yourself as a condition of your staying on the job? (Choose ONE.)

(A) Yes.

(B) No.

(C) Undecided.

4. If the vaccine becomes available but is in short supply, would you demand that you get it for your family/loved ones as a condition of your staying on the job? (Choose ONE.)

(A) Yes.

(B) No.

(C) Not applicable to me; I don’t have family/loved ones here.

(D) Undecided.

Medical First-Responder Group: Session 5

1. The FBI and CDC now believe this disease outbreak is due to terrorism rather than an accidental introduction of the virus into your state. Does the fact that this is terrorism make you more or less afraid of your safety and that of your loved ones? (Choose ONE.)

(A) More afraid.

(B) Less afraid.

(C) Makes no difference.

2. At this point, what is the most likely action you'll take regarding your job? (Choose ONE.)

(A) Stay and work.

(B) Stay away from work by taking sick leave or annual leave.

(C) Take leave without pay.

(D) Quit.

(E) Other (explain briefly).

3. In your own words, briefly describe the kind of information or assurances you would want someone to give you that would help convince you to stay on the job at this time.

4. In your own words, briefly identify who should provide you with that information or those assurances (mentioned in the last question).

5. What role would your spouse/significant other have in helping you make your decision about which choices (see question two, above) you would make regarding your job? (Choose the ONE best answer.)

(A) My spouse/significant other and I would have to agree on the choice; I would take no action regarding my job that my spouse/significant other did not agree with.

(B) My spouse/significant other and I would not have to agree on the choice; I would be willing to take action regarding my job that my spouse/significant other did not agree with.

(C) Not applicable; I don't have a spouse or significant other.

(D) Other (explain briefly)

6. At this point, what actions are you likely to take about your family/loved ones? (Choose the ONE best answer.)

(A Nothing unusual.

(B) Try to restrict their contacts with other people; encourage them to stay more at home, perhaps even keep my kids from school.

(C) Try to get some kind of antibiotics for them and me.

(D) Try to restrict their contacts with other people; encourage them to stay more at home, perhaps even keep my kids from school AND try to get antibiotics for them.

(E) Send my family out of town, to visit friends or relatives elsewhere, but I'll stay home and on the job.

(F) My spouse/significant other can stay here and stay on the job, but I'll leave with my family, and we'll go to visit friends or relatives elsewhere.

(G) Leave with my family, and we'll ALL go to visit friends or relatives elsewhere.

(H) I don't have family or loved ones here, but I would stay in town.

(I) I don't have family or loved ones here, but I would leave town.

(J) Other (explain briefly)

Medical First-Responder Group: Session 6

You hear that there is a possibility that your metropolitan area will be quarantined to prevent anyone infected with the virus from becoming a source of virus for mosquitoes elsewhere in the region. (If the mosquitoes bite a person who is infected, the mosquitoes might then transmit the virus to other people and cattle beyond the area now involved.)

1. If a quarantine was imposed, what would you do about obeying the quarantine? (Choose the ONE best answer.)

(A) I would obey the quarantine and not try to leave the area under any circumstance.

(B) I would obey the quarantine only if it were so heavily enforced that I thought I might be arrested or perhaps even shot if I tried to flee.

(C) I would obey it, but if I really wanted to leave because of business or personal reasons, I would figure some way of getting out.

(D) I would conclude that if the situation had become serious enough for a quarantine, it was time for me to take my family (if I have one) and get out, and nothing would stop me.

(E) Other (explain briefly).

2. Explain briefly in your own words what information or assurances you would want from someone that would make you decide to obey the quarantine.

3. In your own words, briefly describe or identify who should be the source of that information or those assurances (mentioned in the last question).

4. If a quarantine was imposed, and you were called upon to help enforce it, would you do so, or would you try to avoid that duty? (Choose the ONE answer that is closest to your decision.)

(A) Under no circumstances would I report for that kind of duty; I would avoid it, regardless of the circumstances.

(B) I would try to avoid that kind of duty if at all possible, but without breaking any laws or getting myself into trouble.

(C) I would report for duty as directed, without attempting to evade or question the order.

(D) Other (explain briefly)

5. If a quarantine was imposed, and you were called upon to help enforce it, how vigorously would you help enforce it? (Choose the ONE answer that is closest to your decision.)

(A) I would not enforce it at all.

(B) I would help enforce the quarantine but would use my own judgment about whether or not to let individuals pass through.

(C) I would enforce the quarantine as directed, if necessary with the maximum use of force authorized.

(D) Other (explain briefly)

6. At this point, who would you likely consider your most **reliable** source of information about all that has happened thus far?

_______An official from the office of Governor Tom Ridge, at the White House Office of Homeland Security

_______The President of the United States.

_______A medical/science reporter from a major, national newspaper or

TV network.

_______A physician from the CDC in Atlanta, Georgia.

_______A physician from the National Institutes of Health (NIH) in Bethesda, Maryland.

_______A physician from either the CDC or the NIH.

_______The head of the CDC team at the site of the outbreak.

_______My local mayor.

_______An official (but not a physician) from my local Office of Emergency Preparedness.

_______The state health commissioner.

_______A senior public health official from my city.

_______A relative, friend, or colleague who was a physician or at least knew a lot about medicine, or my own personal physician.

_______A trusted friend or loved one who has no special expertise in medicine.

_______I don't know.

_______Other (explain briefly)

7. At this point, who would you consider your least **reliable** sources of information about all that has happened thus far?

_______An official from the office of Governor Tom Ridge, at the White House Office of Homeland Security.

_______The President of the United States.

_______A medical/science reporter from a major, national newspaper or TV network.

_______A physician from the CDC in Atlanta, Georgia.

_______A physician from the National Institutes of Health (NIH) in Bethesda, Maryland.

_______A physician from either the CDC or the NIH.

_______The head of the CDC team at the site of the outbreak.

_______My local mayor.

_______An official (but not a physician) from my local Office of Emergency Preparedness.

_______The state health commissioner.

_______A senior public health official from my city.

_______A relative, friend, or colleague who was a physician or at least knew a lot about medicine, or my own personal physician.

_______A trusted friend or loved one who has no special expertise in medicine.

_______I don't know.

_______Other (explain briefly

8. Who would likely have the greatest **influence** on your personal decisions about what actions you should take, such as staying on the job, evacuating your family, leaving with your family, etc.? (Choose the ONE person who would have the greatest influence.)

(A) An official from the office of Governor Tom Ridge, at the White House Office of Homeland Security.

(B) The President of the United States.

(C) A medical/science reporter from a major, national newspaper or TV network.

(D) A physician from the CDC in Atlanta, Georgia.

(E) A physician from the National Institutes of Health (NIH) in Bethesda, Maryland.

(F) A physician from either the CDC or the NIH.

(G) The head of the CDC team at the site of the outbreak.

(H) My local mayor.

(I) An official (but not a physician) from my local Office of Emergency Preparedness.

(J) The state health commissioner.

(K) A senior public health official from my city.

(L) A relative, friend, or colleague who was a physician or at least knew a lot about medicine, or my own personal physician.

(M) A trusted friend or colleague who has no special expertise in medicine.

(N) Members of my family and/or other loved ones.

(O) I don’t know.

(P) Other (explain briefly)
